# Elevated BTLA Expression Correlates with an Immunosuppressive Microenvironment and Defines Dysfunctional Circulating T Cells in Human Glioblastoma

**DOI:** 10.3390/medsci14040433

**Published:** 2026-07-25

**Authors:** Sanaa Souat, Khadija El Azhary, Sara Bourdoukh, Abdou-samad Kone, Ahmed Qandouci, Zakia Harmak, Khalil Choukri, Abdelhakim Lakhdar, Abdallah Badou

**Affiliations:** 1 Immuno-Genetics and Human Pathology Laboratory, Faculty of Medicine and Pharmacy, Hassan II University of Casablanca, Casablanca 20360, Morocco; sanaa.souat-etu@etu.univh2c.ma (S.S.); elazhary.khadija6m@gmail.com (K.E.A.); sara.bourdoukh-etu@etu.univh2c.ma (S.B.); koneabdousamad@gmail.com (A.-s.K.); ahmedqandouci@gmail.com (A.Q.); zakia.harmak-etu@etu.univh2c.ma (Z.H.); khalil.choukri-etu@etu.univh2c.ma (K.C.); 2 Department of Neurosurgery, University Hospital Center (UHC) Ibn Rochd, Casablanca 20000, Morocco; lakhdar-abdelhakim@hotmail.fr; 3 Laboratory of Research on Neurologic, Neurosensorial Diseases and Handicap, Faculty of Medicine and Pharmacy, Hassan II University of Casablanca, Casablanca 20360, Morocco; 4 City of Innovation and Technology Transfer, Hassan II University of Casablanca, Casablanca 20360, Morocco

**Keywords:** BTLA, glioma microenvironment, immunotherapy, immune checkpoint, immunosuppression, clinical outcome

## Abstract

**Background**: Successful translation of cancer immunotherapy is underscored by the efficacy of PD-1/PD-L1 and CTLA-4 inhibitors in the treatment of various malignancies. However, their limited efficacy in glioma indicates alternative immune escape mechanisms. We investigated B and T Lymphocyte Attenuator (BTLA), a co-inhibitory receptor structurally and functionally analogous to PD-1, to determine if it constitutes a key, unaddressed mechanism of immune escape and a novel therapeutic target in glioma. **Methods**: We analyzed BTLA expression and function within the tumor microenvironment of a Moroccan cohort (*n* = 44). This was complemented by multiparameter flow cytometry on peripheral blood from glioblastoma (GBM) patients (*n* = 8) to assess circulating T cell profiles. Findings were corroborated using independent transcriptomic datasets from TCGA and CGGA cohorts. Single-cell RNA-seq and citeSeq identified specific BTLA-expressing cell populations. **Results:** Elevated BTLA expression was significantly associated with aggressive features and poor overall survival in glioma patients. Mechanistically, BTLA levels were positively correlated with pro-tumorigenic factors, immune infiltration, and immunosuppressive checkpoints. Single-cell and citeSeq analyses revealed that BTLA was primarily expressed by exhausted T cells and conventional type 1 dendritic cells (cDC1) within the GBM microenvironment. Crucially, this phenotype was translated systemically; BTLA defined dysfunctional circulating CD8^+^ and CD4^+^ T cells characterized by diminished IFN-γ production, alongside reduced granzyme B and perforin in CD8^+^ T cells. **Conclusions:** Our findings indicate that BTLA may represent a relevant pathway associated with an immunosuppressive glioma microenvironment. The therapeutic potential of targeting this pathway, particularly in combination with PD-1/PD-L1 blockade, warrants further investigation.

## 1. Introduction

The most common primary malignant tumors of the central nervous system (CNS) in adults are gliomas that arise from glial cells, which are the supporting cells of the brain [1]. Glioblastoma (GBM) is the most lethal and prevalent subtype, representing approximately 14.2% of all tumors and 51% of all malignant tumors [2]. Currently, the median overall survival (OS) for GBM patients ranges between 15 and 23 months [3]. The standard of care consists of maximal safe surgery followed by radiotherapy combined with concurrent and adjuvant temozolomide [4]. Although this regimen has successfully extended patient survival beyond historical benchmarks, notably doubling 2-year survival rates, long-term outcomes remain deeply challenging [5]. Indeed, early disease progression is frequently observed, with the majority of patients succumbing to the tumor within 2 years, and fewer than 6% achieving a 5-year survival milestone [3]. The World Health Organization (WHO) classification of gliomas has been considerably refined. The WHO Central Nervous System 5th edition (CNS5) update in 2021 brought new benefits and useful guidelines to the clinic by updating molecular biomarkers of different tumor types based on the previous histological classification of gliomas from grade 1 to 4 in the 2016 WHO classification [6]. GBMs are now defined as grade 4 with IDH-wildtype.

In parallel with these molecular insights, the glioma tumor microenvironment (TME) is a critical driver of disease progression and treatment resistance [7]. This immunosuppressive niche is fueled by a combination of immunosuppressive cytokines, especially Interleukin-6, Interleukin-10, and Transforming Growth Factor-beta (TGF-β), secreted by tumor cells, microglia, tumor-associated macrophages (TAMs), and regulatory T cells (Treg) [8,9]. These factors collectively suppress T cell activation and proliferation, inhibit natural killer cell activity, and promote the polarization of TAMs towards a pro-tumorigenic M2 phenotype [10]. Two immune checkpoint blockade approaches have been approved by the Food and Drug Administration (FDA). Patients with advanced and recurrent GBM are frequently treated with anti-PD-1 and/or anti-CTLA-4 antibodies, which are often used in combination with chemotherapy or radiation therapy [11]. Despite these treatments being associated with an upregulation of T cells and interferon-γ expression by tumor-infiltrating lymphocytes (TILs), they have largely failed to demonstrate meaningful survival benefits [12,13]. These disappointing outcomes suggest that the PD-1 and CTLA-4 pathways are not the primary, non-redundant drivers of immune evasion in this disease, thereby underscoring the critical need to explore alternative immune checkpoint pathways that may represent novel targets for immunotherapies.

The present study aimed to explore the role of the immune checkpoint BTLA, also known as CD272, in advanced gliomas. BTLA, a member of the CD28 immunoglobulin superfamily (IgSF), is a key regulator of immune activation and inhibition [14]. It is structurally and functionally homologous to established checkpoints, such as CTLA-4 and PD-1 [15]. BTLA is expressed on both lymphoid and myeloid cells, including CD4^+^ and CD8^+^ T cells, B cells, NK cells, macrophages, and dendritic cells [16]. Herpes virus entry mediator (HVEM) is the best-known BTLA ligand. It belongs to the tumor necrosis factor receptor (TNFR) superfamily [17]. BTLA-binding HVEM induces a negative signal that modulates the immune response by inhibiting cell activation, proliferation, and cytokine production [18]. This pathway regulates T-cell reactivity through a dynamic balance of cis and trans interactions. In resting T cells, cis-association of BTLA and HVEM on the same membrane prevents activating NF-κB signaling to maintain baseline tolerance, establishing a co-inhibitory state that functionally dominates over HVEM co-stimulation. Upon T-cell activation, receptor reorganization shifts the axis toward trans-interactions with neighboring cells [15]. Intracellularly, this negative signal is mediated by the phosphorylation of BTLA’s cytoplasmic ITIM/ITSM motifs, which recruit SHP-1/SHP-2 phosphatases to inhibit TCR signaling, although the receptor retains dual signaling potential through a stimulatory Grb2-binding site [15]. Consequently, the BTLA/HVEM axis has been linked to poor prognosis in several solid and hematological tumors [19]. Moreover, aberrant BTLA expression has been found, especially on TILs, and has been linked with negative illness outcomes and suppression of anticancer responses in non-small cell lung carcinoma [20], melanoma [21], gallbladder cancer [22], and hepatocellular carcinoma [23].

Our previous comprehensive immune profiling of glioma patients identified two distinct clusters based on tumor-infiltrating immune cells and immune checkpoint expression [24]. Notably, BTLA was among the most highly expressed inhibitory checkpoints in a cluster defined by poor survival and enriched immunosuppressive infiltrates, including Tregs, M2 macrophages, resting mast cells, and resting NK cells. These findings provide a strong rationale for the present study to investigate the biological functions and clinical relevance of BTLA in gliomas.

## 2. Materials and Methods

### 2.1. Clinical Samples

BTLA expression was evaluated in 44 tumor samples from glioma patients recruited from the Neurosurgery Department of Ibn Rochd University Hospital. All glioma tissues were classified according to the 2016 World Health Organization (WHO) guidelines: 14 specimens were grade 1, five were grade 2, three were grade 3, and 22 were grade 4 (refer to Supplementary Table S1 for clinicopathological parameters). All selected patients had a prior glioma diagnosis and had not received any treatment before undergoing tumor resection. The inclusion criteria were provision of informed consent to participate in the study and complete study documentation. Patients were excluded if they had incomplete documentation or lacked informed consent. Clinical information, including age, sex, IDH status, histological type, and grade, was obtained from the patients’ medical records. Additionally, for functional assays using flow cytometry, fresh venous blood was collected from eight IDH-wildtype grade 4 GBM patients, following written informed consent obtained in accordance with the regulations approved by the ethics committee.

### 2.2. Total RNA Isolation and cDNA Synthesis

Total RNA was extracted from 44 fresh biopsy samples using TRIzol reagent (Invitrogen, Massy, France) according to the manufacturer’s instructions. RNA concentration and purity were measured using a BioDrop μLite spectrophotometer (Fisher Scientific, Illkirch-Graffenstaden, France). cDNA synthesis was performed with 0.5 μg of total RNA in a 20 μL reaction volume containing 1 μL Random Hexamer Primer (25 µg; Bioline, Livron-sur-Drôme, France), 0.5 μL of RNase Inhibitor (Invitrogen, France), 0.5 μL of Tetro Reverse Transcriptase Enzyme (Bioline, Livron-sur-Drôme, France), 4 μL of dNTP (10 mM), and 4 μL of Tetro Reverse Transcriptase buffer. The final volume was adjusted by adding RNase-free water. The reaction mix was incubated at 25 °C for 10 min, followed by 42 °C for 45 min, and 70 °C for 15 min, and held at 4 °C.

### 2.3. Real-Time q-PCR Assay

Relative gene expression was quantified using real-time PCR in the presence of the fluorescent dye SYBR^TM^ Green PCR Master MIX (Thermo Fisher Scientific, Waltham, MA, USA) on a croBEE^®^ real-time PCR detector system. PCR assay was performed with specific primer pairs for BTLA and β-actin at 10 µM in a final reaction volume of 20 µL. The Mix contained 10 µL SYBR^TM^ Green, 0.5 µL each of forward and reverse primers, 2 µL cDNA, and 7 µL of nuclease-free water. Each run included a no-template control (NTC) with water substituted for cDNA. The thermal cycling protocol consisted of an initial polymerase activation at 95 °C for 10 min, then 40 cycles of denaturation at 95 °C for 15 s, and annealing/extension at 60 °C for 60 s. A melting curve analysis was performed post-amplification to confirm reaction specificity. β-actin was used as an endogenous control to normalize BTLA expression levels in glioma samples. Ct values were converted into relative quantification using the 2^−ΔCt^ (ΔCt = Ct BTLA gene − Ct β-actin) method. The primer pairs used were as follows:

- β-Actin Forward Primer: 5′-GAGATGGCCACGGCTGCTT-3′
- β-Actin Reverse Primer: 5′-GCCACAGGACTCCATGCCCA-3′
- BTLA Forward Primer: 5′-ACCCTCCAAGGACGAAATG-3′
- BTLA Reverse Primer: 5′-TTAATTTCCCTTCCTGCTGTG-3′
- PD-1 Forward Primer: 5′-GCTGGATTTCCAGTG-3′
- PD-1 Reverse Primer: 5′-ATGAGGTGCCCATTC-3′

### 2.4. PBMC Preparation and Cell Culture

Freshly isolated peripheral blood mononuclear cells (PBMCs) were obtained by density gradient centrifugation using Ficoll-Histopaque (Sigma, Saint-Louis, MO, USA) at 1000 g for 20 min at 20 °C. The PBMC layer was then collected, washed twice with NaCl (0.9%) at 400 g for 10 min, and resuspended in complete RPMI-1640 medium (Sigma, Saint-Louis, MO, USA) containing 10% fetal bovine serum (FBS) (Sigma, Saint-Louis, MO, USA) and 1% penicillin-streptomycin (Gibco, Life Technologies, Carlsbad, CA, USA). For flow cytometry analysis of IFN-γ production, PBMCs were seeded into 96-well plates at a concentration of 1 × 10^6^ cells/mL per well. Cells were activated using 2.5 µg/mL plate-bound human anti-CD3 (clone OKT3, GeneTex, Irvine, CA, USA) and 1.25 µg/mL soluble anti-CD28 (clone CD28.2, GeneTex, Irvine, CA, USA) in complete RPMI medium, followed by overnight incubation. Prior to staining, Brefeldin A (Invitrogen, Eugene, OR, USA) was added to cultured cells at a final concentration of 10 µg/mL for less than 12 h.

### 2.5. Flow Cytometry

To detect IFN-γ production in BTLA^+^CD4^+^, BTLA^−^CD4^+^, BTLA^+^CD8^+^, and BTLA^−^CD8^+^ T cell subsets, the following monoclonal antibodies were used: CD4-PE/Cy7 (Clone RFT4; SouthernBiotech, Birmingham, AL, USA); CD8-APC (Clone RPA-T8; MBL, Japan, Tokyo), BTLA-PE (Clone J168-540, BD Pharmingen, San Jose, CA, USA), and IFN-γ-FITC (Clone B27; ORIGENE, Rockville, MD, USA), along with appropriate isotype controls. For intracellular staining, cells were fixed with 4% formaldehyde and permeabilized with 0.1% Triton X-100. For the detection of perforin and granzyme B expression levels in BTLA^+^CD8^+^ and BTLA^−^CD8^+^ populations, the following monoclonal antibodies were used: CD8-APC (Clone RPA-T8; MBL, Japan, Tokyo), CD3-PerCPCy5.5 (Clone OKT3, Invitrogen, Eugene, OR, USA), BTLA-PE (Clone J168-540, BD Pharmingen, San Jose, CA, USA), perforin-FITC (B-D48, Biorbyt, Cambrige, Cambridgeshire, UK), and granzyme B-FITC (Clone 3H2182 (GB11), USBiological life Sciences, Salem, MA, USA). Appropriate isotype controls were also included. Cells were then fixed and permeabilized as described for IFN-γ staining. Samples were acquired on a BD FACSLyric^TM^ cytometer, and data were analyzed using Kaluza 2.3 software. Photomultiplier tube (PMT) voltages were set using BD CS&T beads (BD Biosciences, San Jose, CA, USA).

### 2.6. Data Acquisition and Preprocessing

RNA-seq data and associated clinical information for glioma patients were acquired from two cohorts. The Cancer Genome Atlas (TCGA) Firehose Legacy cohorts, comprising 515 low-grade glioma and 152 GBM, were acquired from an open access database https://www.cbioportal.org/ (accessed on 19 December 2024). To address the molecular heterogeneity of gliomas, we restricted all analyses to three molecularly homogeneous groups based on the WHO classification: GBM, grade 4, IDH-wildtype; Astrocytoma, IDH-mutant; and Oligodendroglioma. The final analytical cohort (*n* = 214) consisted of three distinct WHO-defined groups. An independent validation cohort of 693 patients was obtained from the Chinese Glioma Genome Atlas (CGGA), which is publicly available at http://www.cgga.org.cn/ (accessed on 13 November 2024). Applying identical inclusion criteria to the CGGA dataset yielded a final validation cohort (*n* = 309) restricted to the same three molecularly defined subgroups. All cases were selected based on the availability of complete transcriptomic data and matched clinicopathological parameters. Expression profiles for TCGA and CGGA datasets were log2-transformed (TPM+1 and FPKM+1) for all downstream analyses.

### 2.7. Evaluation of Tumor-Infiltrating Immune Cells

To estimate the relative proportions of tumor-infiltrating immune cell types (TIICs), a computational deconvolution approach was performed. The BioBean platform https://sxdyc.ezygene.com/singleCollectionTool (accessed on 15 February 2025) was used to perform comprehensive immune landscape analysis using three distinct methods: ImmuCellAI to evaluate the relative abundance of 24 immune cell subpopulations between high- and low-BTLA phenotypes; the ESTIMATE algorithm to compute stromal, immune, and estimate scores along with tumor purity to characterize the tumor microenvironment; and the TIDE algorithm to quantify M2-polarized tumor-associated macrophage (TAM.M2) infiltration and interferon (IFN) signature expression.

### 2.8. Biological Function and Gene Set Enrichment Analysis

To identify signaling pathways and biological processes differentially enriched between high- and low-BTLA expression groups based on median expression, we performed gene set enrichment analysis using GSEA (v4.3.2) software https://www.gsea-msigdb.org/ (accessed on 15 May 2025). The TCGA and CGGA datasets were normalized using the DESeq2 method prior to analysis. Standard GSEA was performed using an expression matrix (.gct file) and a phenotype file (.cls file), defining the BTLA-high and BTLA-low groups, and using the signal2Noise ranking metric. We exploited the Molecular Signatures Database (MSigDB) collections, including Gene Ontology biological processes (GO: BP) and hallmark gene sets, using the chip annotation file MSigDB (v2025.1 Hs.chip). Statistical significance was established using 1000 permutations with dual thresholds: nominal *p*-value less than 0.05, and False Discovery Rate (FDR) below 0.25.

### 2.9. Single-Cell Resolution Analysis

Single-cell analysis of BTLA expression was performed using the GBM-GSE131928_Smartseq2 dataset from the Tumor Immune Single-cell Hub 2 (TISCH2) database http://tisch.comp-genomics.org/ (accessed on 29 April 2026). The expression of BTLA, CD160, TNFSF14 (LIGHT), and TNFRSF14 (HVEM) across stromal, immune, and malignant cells was evaluated and visualized using violin plots. Additionally, the expression of exhaustion markers PDCD1 (PD-1), LAG3, and HAVCR2 (TIM3) was assessed across major cell lineages. To further evaluate BTLA expression in myeloid populations, Uniform Manifold Approximation and Projection (UMAP) for dimensionality reduction was applied to two datasets from the Brain Immune Atlas https://www.brainimmuneatlas.org/ (accessed on 29 April 2026): the Human GBM TAM, DCs, and Monocytes citeSeq dataset and the Human GBM DCs citeSeq dataset. Differentially expressed genes (DEGs) were downloaded from the TISCH2 database for the GSE131928_Smartseq2 dataset. Differential cell surface protein expression (ADT) results for citeSeq-derived GBM TAM, monocyte, and DC subpopulations were obtained from the Brain Immune Atlas database.

### 2.10. Statistical Analysis

All statistical analyses, including data visualization and heatmap generation, were performed using GraphPad Prism (v8.0.1) and RStudio (v4.3.1). The Shapiro–Wilk and Kolmogorov–Smirnov normality tests were used to evaluate the distribution of the data. Comparisons of data between or among groups were performed using the Wilcoxon test, Mann–Whitney test, and Kruskal–Wallis test, followed by Dunn’s multiple comparison test. Welch’s *t*-test was applied to compare mean expression, accounting for the standard deviations and sample sizes of each group. Spearman’s non-parametric test was used to estimate the correlation coefficients. The effect size was reported as r. Multiple testing correction was performed using the Benjamini–Hochberg (FDR) method when applicable. Kaplan–Meier survival curves and the log-rank test were used to evaluate the significance of survival time differences by “survival” and “survminer” R packages (v2024.04.2+764). The surv_cutpoint function, which is based on maximally selected rank statistics (Maxtat R package), was used to identify the optimal cutoffs. These thresholds were further validated by 1000 bootstrap iterations, with the final cutoffs defined as the median of bootstrap estimates with 95% CI. This approach was applied identically to both TCGA and CGGA datasets, ensuring that the classification was cohort-independent and statistically robust. Accordingly, patients within each dataset were classified into BTLA-low or BTLA-high and PD-1-low or PD-1-high groups. Multivariate Cox regression analyses were carried out to identify overall survival (OS) related clinical characteristics. All two-tailed *p*-values less than 0.05 were considered statistically significant.

### 2.11. Ethics Approval and Participation Consent

The study protocol was reviewed and approved by the Ethics Committee of Ibn Rochd University Hospital in Casablanca. Prior to inclusion in the study, written informed consent was obtained from all adult patients and from the parents or legal guardians of participants under the age of 18.

## 3. Results

### 3.1. BTLA Gene Expression Was Associated with Aggressive Clinicopathological Features in Glioma Patients

**Figure 1:**
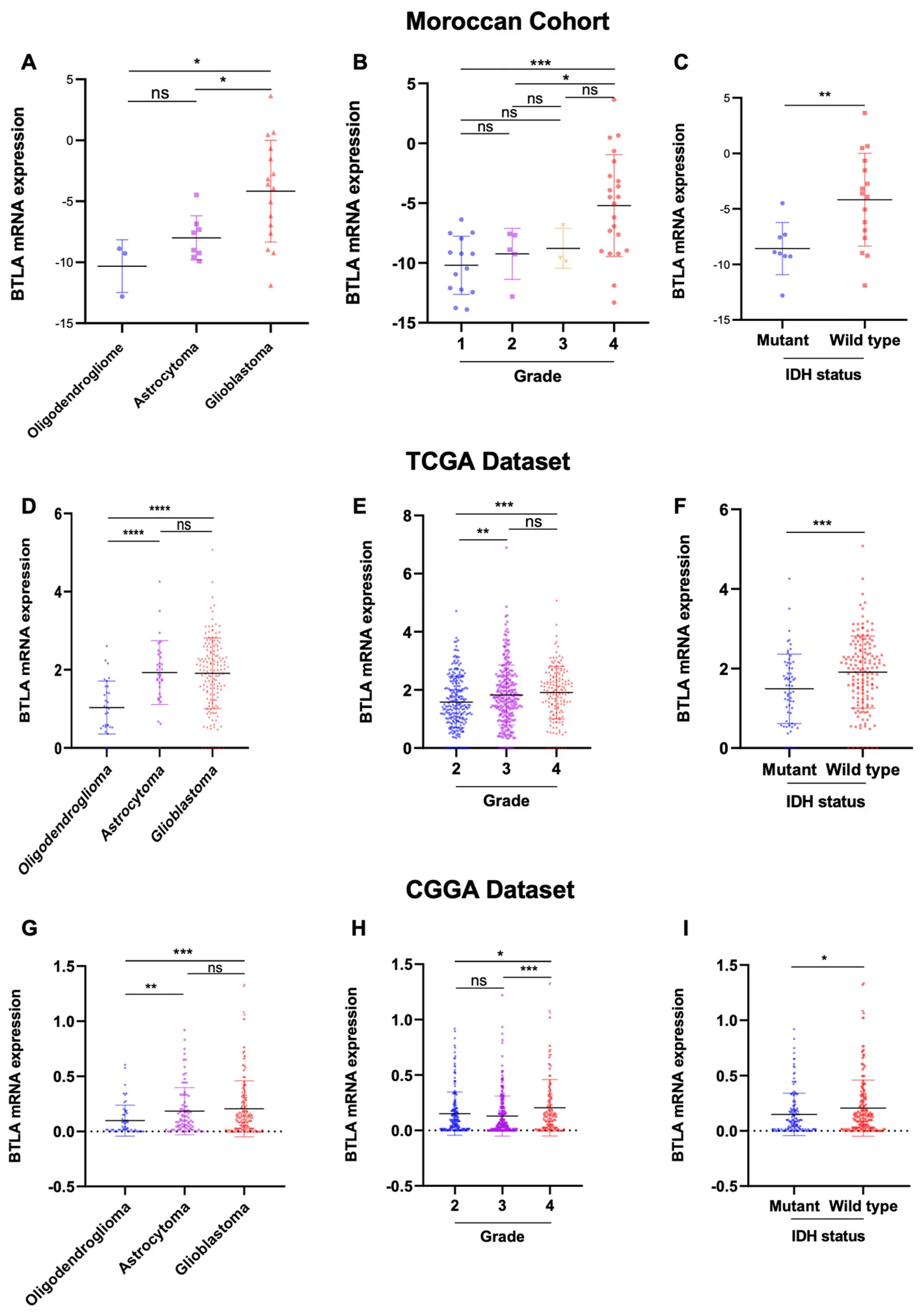
BTLA gene expression was associated with aggressive clinicopathological features in gliomas across the Moroccan cohort, TCGA, and CGGA datasets. (**A**,**D**,**G**) BTLA mRNA expression across the (**A**) Moroccan (*n* = 27), (**D**) TCGA (*n* = 215), and (**G**) CGGA (*n* = 332) cohorts was significantly enriched in GBM IDH-wildtype compared to lower-grade oligodendroglioma. (**B**,**E**,**H**) Distribution of BTLA expression levels according to WHO tumor grade. In the (**B**) Moroccan (*n* = 44), (**E**) TCGA (*n* = 666), and (**H**) CGGA (*n* = 633) cohorts, BTLA levels significantly increase with advancing tumor grade. (**C**,**F**,**I**) Comparative analysis of BTLA gene expression based on IDH status in the (**C**) Moroccan (*n* = 24), (**F**) TCGA (*n* = 215), and (**I**) CGGA (*n* = 332) datasets. IDH-Wildtype tumors consistently exhibit significantly higher BTLA levels across all cohorts. Moroccan cohort data are represented as log2 (2^−ΔCt^). TCGA and CGGA data were log2-transformed using TPM+1 and FPKM+1 normalization, respectively. Group comparisons were performed using the Mann–Whitney test (**A**–**I**). A two-tailed *p*-value < 0.05 was considered significant, ns, not significant; * *p*-value < 0.05; ** *p*-value < 0.01; *** *p*-value < 0.001; **** *p*-value < 0.0001.

To investigate the association pattern and assess the potential role of BTLA in human glioma, we initially analyzed 44 glioma tissues from Moroccan patients and measured BTLA levels using RT-qPCR (represented as log2 (2^−ΔCt^) values). Patient clinical parameters are summarized in Supplementary Table S1. We first evaluated the expression of BTLA and its association with key clinical features of gliomas. BTLA expression was significantly higher in patients aged > 45 years (*p* = 0.0252) but showed no significant association with sex (*p* = 0.6291) (Supplementary Table S1). Analysis of histological subtypes revealed a significant upregulation of BTLA in GBM, IDH-wildtype (*n* = 16), the most aggressive subtype, compared to astrocytoma (*n* = 8, *p* = 0.0159) and oligodendroglioma (*n* = 3, *p* = 0.0227) (Figure 1A). Furthermore, BTLA expression was highly enriched in WHO grade 4 tumors (*n* = 22) compared to grades 1 (*n* = 14, *p* = 0.0002) and 2 (*n* = 5, *p* = 0.0397) (Figure 1B). Consistently, BTLA levels were significantly elevated in IDH-wildtype gliomas (*n* = 16) compared to IDH-mutated gliomas (*n* = 8, *p* = 0.0265) (Figure 1C). To corroborate our initial findings, we extended our analysis to two large independent cohorts, TCGA and CGGA (Supplementary Table S2). These datasets confirmed that BTLA expression was significantly higher in GBM IDH-wildtype (TCGA: *n* = 152; CGGA: *n* = 190) than in oligodendroglioma (TCGA: *n* = 31; CGGA *n* = 58) (*p* < 0.0001 and *p* < 0.001, respectively) (Figure 1D,G). Regarding tumor grade, BTLA expression was significantly higher in grade 4 (*n* = 152) than in grade 2 (*n* = 249) in TCGA (*p* = 0.0001, Figure 1E) and in grade 4 (*n* = 190) than in grade 2 (*n* = 188) in CGGA (*p* = 0.0008, Figure 1H). Finally, the association with IDH status was validated globally; IDH-wildtype patients exhibited significantly elevated BTLA expression compared to IDH-mutant patients in both TCGA (*n* = 152 vs. 63, *p* = 0.0009) and CGGA (*n* = 190 vs. 142, *p* = 0.0239) (Figure 1F,I). Collectively, these results consistently demonstrated that elevated BTLA expression was associated with aggressive clinicopathological features in patients with glioma.

### 3.2. Elevated BTLA Expression in the Glioma Microenvironment Was Associated with Poor Overall Survival

**Figure 2:**
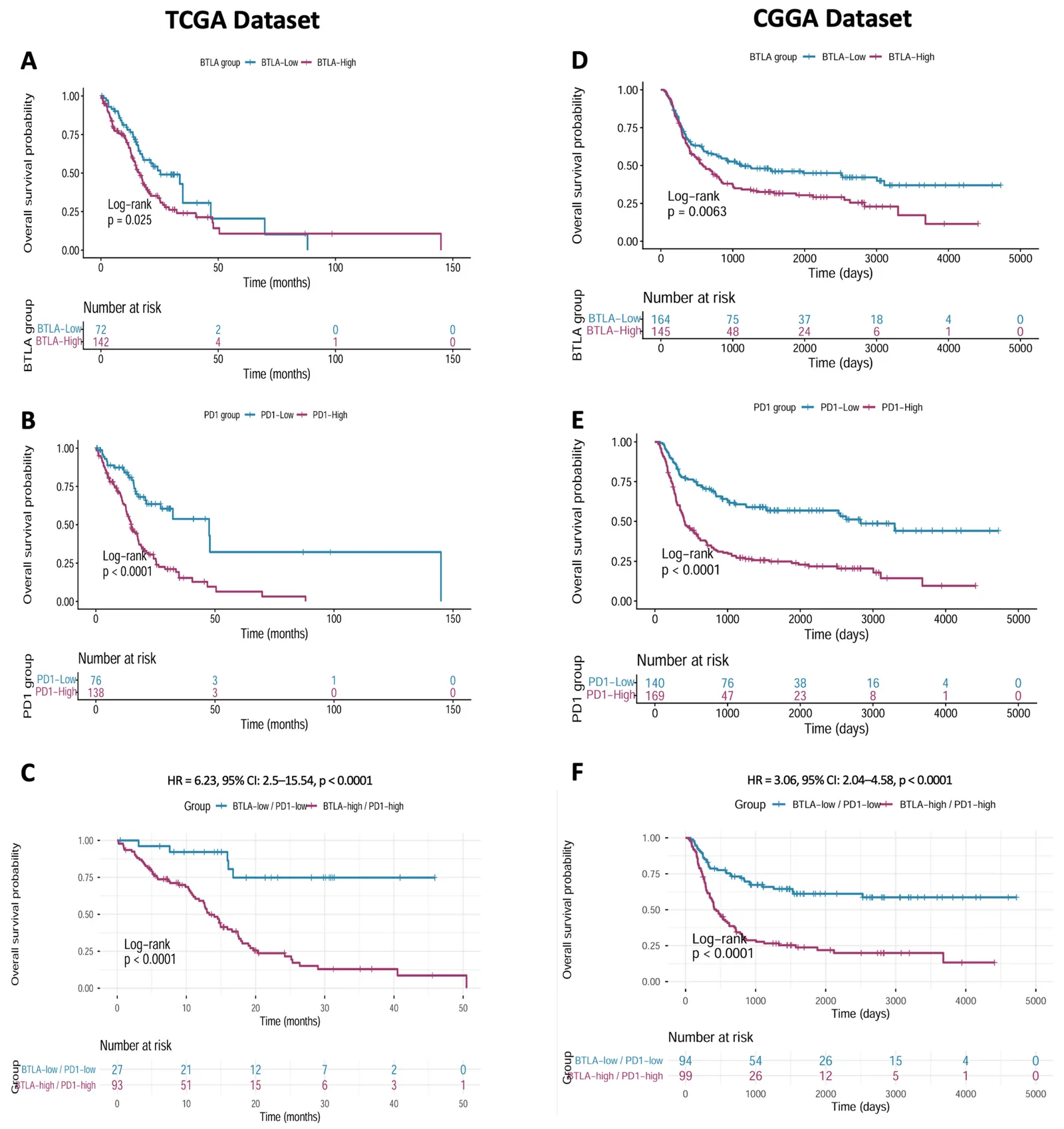
Overexpression of BTLA was associated with poor survival in glioma patients from TCGA and CGGA cohorts. (**A**,**D**) Kaplan–Meier survival curves revealing that high BTLA expression was significantly associated with reduced overall survival (OS) in both (**A**)TCGA (HR = 1.55, 95% CI: 1.05–2.29, *p* = 0.025) and (**D**) CGGA (HR = 1.48, 95% CI: 1.12–1.97, *p* = 0.0063) datasets. (**B**,**D**) High PD-1 expression correlates with diminished survival outcomes in glioma patients within the (**B**) TCGA (HR = 2.87, 95% CI: 1.85–4.43, *p* < 0.0001) and (**E**) CGGA (HR = 2.60, 95% CI: 1.92–3.53, *p* < 0.0001) cohorts. (**C**,**F**) Simultaneous high expression of both BTLA and PD-1 (double-high) correlates with the poorest overall survival compared to double-low in (**C**) TCGA (HR = 6.23, 95% CI: 2.5–15.54, *p* < 0.0001) and (**F**) CGGA (HR = 3.06, 95% CI: 2.04–4.58, *p* < 0.0001). Patients were classified into low and high expression groups based on an optimal bootstrap-validated cutoff. Risk tables indicate the number of patients at risk at each time point. Prognostic significance was assessed by the Log-rank test using a Kaplan–Meier plot. *p*-value less than 0.05 was considered statistically significant.

Having established that elevated BTLA expression was a consistent marker of aggressive clinicopathological features, we investigated its possible impact on patient outcomes. Since high tumor grade and IDH-wildtype status are established predictors of unfavorable outcomes, and given that the PD-1 pathway represents a heavily investigated yet therapeutically challenging component in glioma, we sought to determine the individual and comparative prognostic value of BTLA and PD-1. Kaplan–Meier survival analysis revealed that high BTLA expression was significantly associated with decreased overall survival (OS) in both TCGA (HR = 1.55, 95% CI: 1.05–2.29, *p* = 0.025) and CGGA (HR = 1.48, 95% CI: 1.12–1.97, *p* = 0.0063) datasets (Figure 2A,D). Similarly, high PD-1 expression was associated with poor prognosis in these cohorts (TCGA: HR = 2.87, 95% CI: 1.85–4.43, *p* < 0.0001; CGGA: HR = 2.60, 95% CI: 1.92–3.53, *p* < 0.0001) (Figure 2B,E).

Most strikingly, patients characterized by the co-expression of both BTLA and PD-1 exhibited the worst clinical outcomes. The hazard ratio for the double-high group reached 6.23 in the TCGA cohort (95% CI: 2.5–15.54, *p* < 0.0001) and 3.06 in the CGGA cohort (95% CI: 2.04–4.58, *p* < 0.0001) when compared to the double-low group (Figure 2C,F). These results indicate that BTLA and PD1 were cooperatively associated with poor prognosis in patients with glioma.

To determine whether the prognostic value of BTLA is independent of established clinical variables, we performed a multivariate Cox regression analysis. After deleting samples with incomplete clinical data, the final analysis included 276 patients from TCGA and 609 from CGGA cohorts. After adjusting for potential confounders, including age, tumor grade, IDH mutation status, and sex, BTLA remained a significant independent prognostic factor in the larger CGGA cohort (HR = 1.7, 95% CI: 1.24–2.3, *p* < 0.001). However, in the TCGA cohort, BTLA did not reach statistical significance as an independent factor (HR = 1.2, 95% CI: 0.85–1.8, *p* = 0.267) (Supplementary Figure S1A,B). These findings suggest that while BTLA was a potent indicator of poor survival, its prognostic independence may be cohort-dependent and was likely influenced by the strong confounding effects of IDH status and tumor grade, particularly within the smaller TCGA dataset.

To further strengthen this analysis, we performed separate multivariate analyses evaluating PD-1 alone as well as the combined BTLA/PD-1 groups. PD-1 emerged as a significant independent prognostic factor in the CGGA cohort (HR = 2.0, 95% CI: 1.10–2.00, *p* = 0.01) but failed to reach significance in the TCGA cohort (HR = 1.1, 95% CI: 0.80–2.00, *p* = 0.49) (Supplementary Figure S2A,B). Furthermore, the double-high BTLA/PD-1 group in only the CGGA cohort maintained a highly significant independent survival disadvantage compared to the double-low reference group (HR = 3.0, 95% CI: 1.5–7.0, *p* = 0.002) (Supplementary Figure S3A,B). Taken together, these results demonstrate the cooperative prognostic value of these two immune checkpoints, highlighting BTLA as a potent marker of aggressive disease and a promising candidate for dual checkpoint-targeted therapies.

### 3.3. BTLA Expression Was Correlated with a Broad Immunosuppressive Checkpoint Profile in Human Gliomas

**Figure 3:**
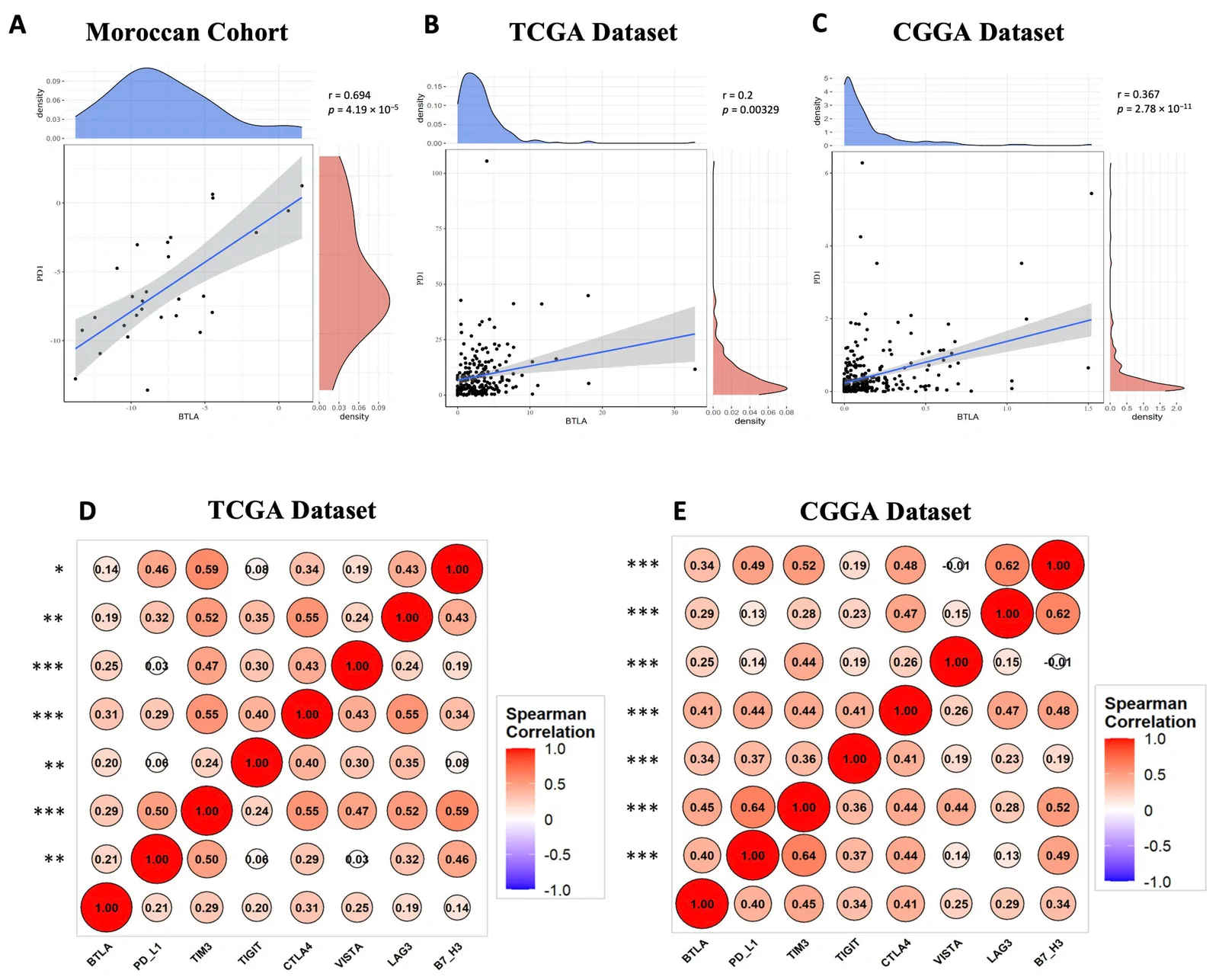
BTLA expression correlated with key immune checkpoint regulators in glioma. (**A**–**C**) BTLA demonstrates a significant positive correlation with the inhibitory immune checkpoint PD-1 across Moroccan (*n* = 28, r = 0.694, *p* = 4.19 × 10^−5^, 95% CI: 0.35–0.82), TCGA (*n* = 214, r = 0.2, *p* = 0.00329, 95% CI: 0.06–0.32), and CGGA (*n* = 309, r = 0.367, *p* = 2.78 × 10^−11^, 95% CI: 0.27–0.46) cohorts. (**D**,**E**) Correlation matrices between BTLA and a panel of immune checkpoint markers in gliomas based on TCGA (*n* = 214) and CGGA (*n* = 309) datasets. Circle size is proportional to −log10(p(FDR)), where larger circles indicate stronger statistical significance after FDR correction. Color intensity represents Spearman’s correlation coefficient: red indicates a positive correlation; blue indicates a negative correlation. Significance levels (FDR-adjusted): * *p* < 0.05; ** *p* < 0.01; *** *p* < 0.001.

The potent association of both BTLA and PD-1 with poor survival established their significant prognostic value and prompted us to investigate their potential interactions. First, we assessed the correlation between BTLA and PD-1 expression across three independent cohorts: Moroccan (*n* = 28), TCGA (*n* = 214), and CGGA (*n* = 309). We observed a significant positive correlation in all three datasets: the Moroccan cohort (r = 0.694, 95% CI: 0.35–0.82, *p* = 4.19 × 10^−5^), TCGA cohort (r = 0.2, 95% CI: 0.06–0.32, *p* = 0.00329), and CGGA cohort (r = 0.367, 95% CI: 0.27–0.46, *p* = 2.78 × 10^−11^), suggesting a consistent co-regulatory link (Figure 3A–C).

To delve deeper into the underlying immune landscape, we investigated the interrelationships between BTLA and a broader spectrum of checkpoint regulators. Beyond PD-1, BTLA expression demonstrated strong positive correlations with several key inhibitory receptors and ligands, including PD-L1, TIM3, TIGIT, CTLA-4, VISTA, LAG3, and B7-H3, across both TCGA and CGGA datasets (Figure 3D,E). Collectively, the synchronous expression of BTLA with an extensive panel of inhibitory molecules suggests a coordinated mechanism of immune evasion in glioma.

### 3.4. BTLA Gene Expression Was Linked to Specific Immune Infiltrates and Immunosuppressive Features

The malignant progression of glioma is heavily influenced by its immunosuppressive microenvironment. As our data linked high BTLA expression to aggressive clinicopathological features and poor survival, we hypothesized that BTLA is associated with the enrichment or altered composition of specific immune cell populations. We employed the Immune Cell Abundance Identifier (ImmuCellAI) algorithm to estimate the abundance of 24 immune subsets within the tumor microenvironment (TME).

**Figure 4:**
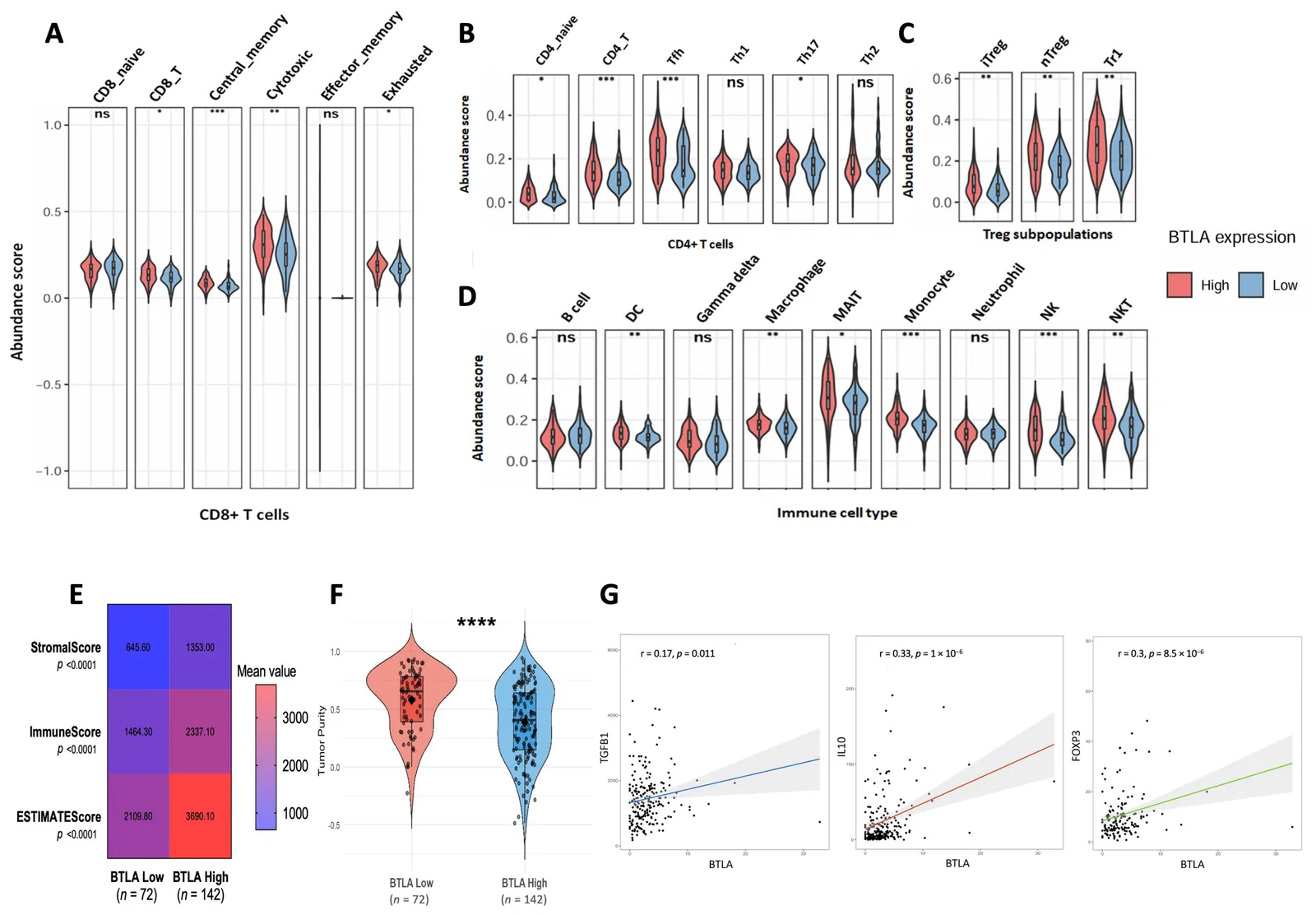
Association between BTLA expression and tumor infiltration in the TCGA dataset. (**A**–**D**) High BTLA expression was associated with a specific immune infiltration profile. Immune cell abundance: Violin plots illustrating immune cell distributions analyzed using the ImmuCellAI algorithm, comparing BTLA-high (*n* = 142, red) and BTLA-low (*n* = 72, blue) phenotypes across (**A**) CD8^+^ T cells, (**B**) CD4^+^ T cells, (**C**) Treg subpopulations, and (**D**) general immune populations. (**E**) BTLA-high tumors were linked to increased overall immune and stromal cell infiltration. Heatmap of tumor microenvironment scores according to BTLA expression status using the ESTIMATE algorithm. Data are presented as mean values per group: BTLA-low (*n* = 72), BTLA-high (*n* = 142). Values represent raw means, and color denotes Z-score transformation (red: above mean; Blue: below mean). All scores were significantly elevated in the BTLA-high group (*p* < 0.0001) (**F**) Elevated BTLA expression was associated with reduced tumor purity. Violin plots showing that BTLA-high tumors exhibited significantly lower tumor purity compared to the BTLA-low group (*p* < 0.0001). (**G**) BTLA expression was positively correlated with critical immunosuppressive cytokines and regulatory markers. Spearman correlation plots demonstrating positive correlation between BTLA expression and TGFβ (r = 0.17, *p* = 0.011), IL10 (r = 0.33, *p* = 1 × 10^−6^), and Foxp3 (r = 0.3, *p* = 8.5 × 10^−6^). Group comparisons were performed using the Mann–Whitney test (**A**–**D**,**F**) or Welch’s *t*-test (**E**). A two-tailed *p*-value < 0.05 was considered significant, ns, not significant; * *p* < 0.05; ** *p* < 0.01; *** *p* < 0.001; **** *p* < 0.0001.

In the TCGA cohort, the BTLA-high phenotype was characterized by a distinct pattern of immune infiltration. Within the CD8^+^ T cell compartment, we observed significant enrichment of the central memory, cytotoxic, and exhausted subsets (Figure 4A). Furthermore, the BTLA-high TME was heavily infiltrated by innate immune cells, including dendritic cells, macrophages, MAIT cells, monocytes, NK cells, and NKT cells (Figure 4D), alongside various adaptive CD4^+^ T helper lineages, such as Th17 and follicular helper T (Tfh) cells (Figure 4B). Notably, a critical finding was the significant enrichment of multiple regulatory T cell subpopulations, including type 1 regulatory T cells (Tr1), natural Tregs (nTregs), and induced Tregs (iTregs) (Figure 4C).

These findings were largely recapitulated in the CGGA cohort, although with some cohort-specific nuances. In the CGGA analysis, while the overall immunosuppressive trend remained, we observed lower infiltration levels of Th1 cells and neutrophils and a relative increase in CD8 naïve and gamma delta T cells compared to the TCGA cohort (Supplementary Figure S4A–D).

Consistent with this cellular landscape, the ESTIMATE algorithm revealed that BTLA-high tumors across both cohorts possessed significantly higher levels of non-malignant cell infiltration. Specifically, Immune scores were substantially elevated in the BTLA-high phenotype (TCGA: β = 872.9, 95% CI: 482.5–1263.2, *p* < 0.0001; CGGA: β = 1014.2, 95% CI: 636.3–1392.1, *p* < 0.0001), as were Stromal scores (TCGA: β = 707.4, 95% CI: 401.7–1013.2, *p* < 0.0001; CGGA: β = 687.6, 95% CI: 369.7–1005.5, *p* < 0.0001). Consequently, the overall ESTIMATE scores showed a dramatic increase (TCGA: β = 1580.3, 95% CI: 907.6–2253, *p* < 0.0001; CGGA: β = 1701.8, 95% CI: 1025.6–2378, *p* < 0.0001), which directly corresponded to a significant reduction in tumor purity (TCGA and CGGA: *p* < 0.0001) (Figure 4E,F, and Supplementary Figure S4E,F).

Finally, BTLA expression demonstrated a significant positive correlation with the regulatory T cell marker Foxp3 (TCGA: r = 0.3, *p* = 8.5 × 10^−6^; CGGA: r = 0.22, *p* = 7.1 × 10^−5^) and key immunosuppressive cytokines, including TGFB1 (TCGA: r = 0.17, *p* = 0.011; CGGA: r = 0.37, *p* = 1.5 × 10^−11^) and IL10 (TCGA: r = 0.33, *p* = 1 × 10^−6^; CGGA: r = 0.34, *p* = 5.4 × 10^−10^) (Figure 4G and Supplementary Figure S4G).

**Figure 5:**
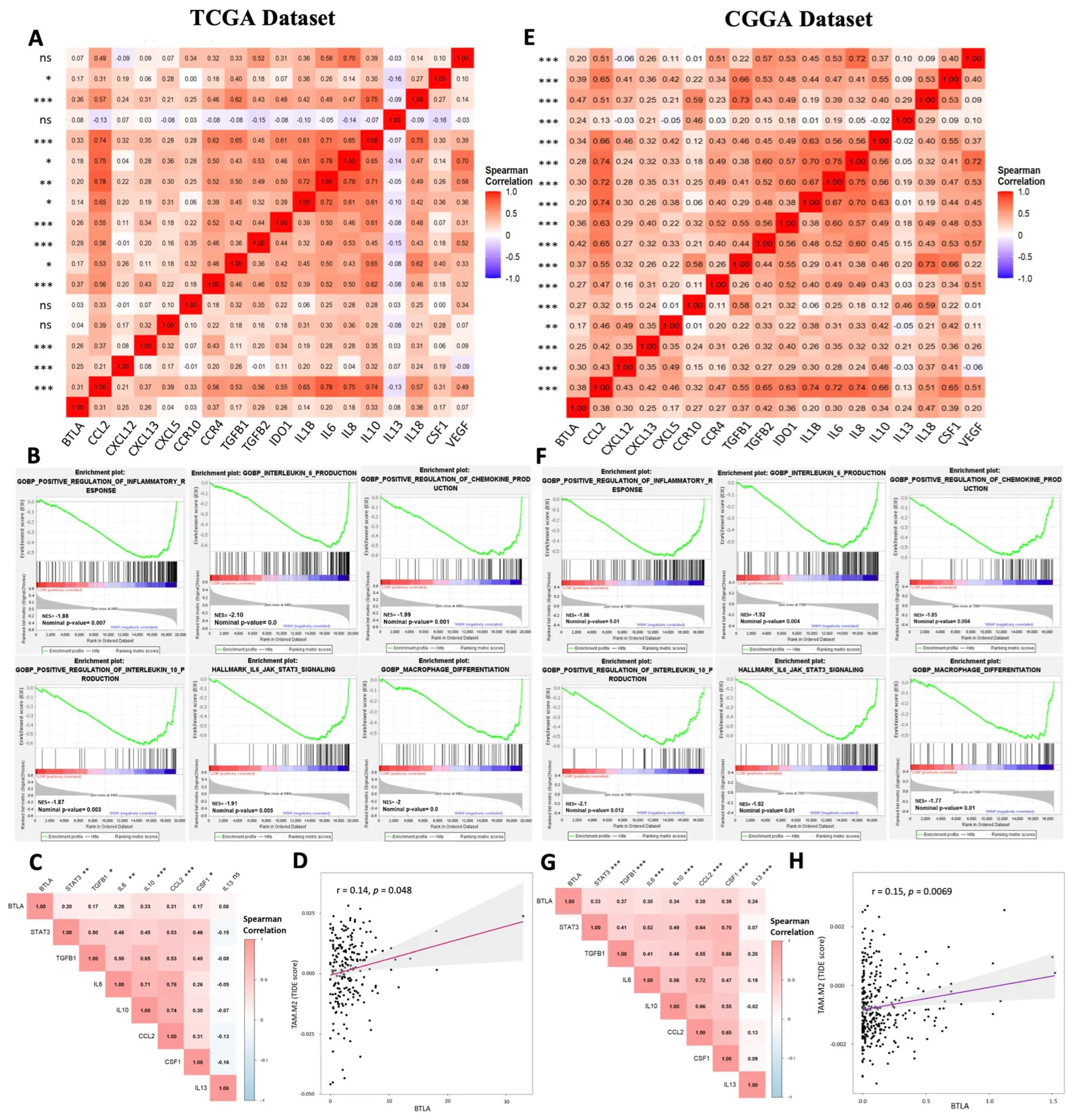
BTLA expression correlated with immunosuppressive and pro-tumorigenic factors. (**A**,**E**) BTLA expression was positively associated with an immunosuppressive tumor microenvironment. Spearman correlation heatmaps illustrating the relationship between BTLA mRNA expression and a panel of immunosuppressive chemokines, cytokines and pro-tumorigenic factors in the (**A**) TCGA (*n* = 214) and (**E**) CGGA (*n* = 309) datasets. Color intensity and numerical value reflect the strength of positive (red) or negative (blue) correlations. (**B**,**F**) High BTLA expression was significantly enriched in immunosuppressive biological processes and hallmark pathways. Enrichment plots demonstrating significant biological processes and immune pathway-related hallmark genes enriched in the high-BTLA phenotype. The ranking metric measures a gene’s correlation with a phenotype. The value of the ranking metric goes from positive to negative as you move down the ranked list. A positive value indicates correlation with the first phenotype (Low-BTLA group, red color) and a negative value indicates correlation with the second phenotype (High-BTLA group, blue color). (**C**,**G**) BTLA expression was positively correlated with key molecular drivers of pro-tumor M2 macrophage polarization. Correlation matrices highlighting the association between BTLA and critical drivers of M2-type macrophage differentiation factors. Red intensity denotes increasing correlation strength. (**D**,**H**) BTLA expression was correlated with increased TAM.M2 macrophage infiltration in tumors. Scatter plots showing a significant positive correlation between BTLA expression and the M2 macrophage abundance score in the (**D**) TCGA (r = 0.14, *p* = 0.048) and (**H**) CGGA (r = 0.15, *p* = 0.0069). Correlation coefficients were determined by Spearman correlation test. GSEA (v4.3.2) was performed using DESeq2-normalized TCGA (*n* = 667) and CGGA (*n* = 693) datasets, stratified by median BTLA expression. Significance levels (FDR-adjusted): * *p* < 0.05; ** *p* < 0.01; *** *p* < 0.001. GSEA significance was established with a nominal *p* < 0.05 and FDR < 0.25.

To define the functional role of BTLA in shaping the immunosuppressive tumor microenvironment, we profiled the expression of pivotal molecular mediators involved in immune evasion and tumor progression. Our analysis revealed that BTLA expression was positively correlated with a network of immunosuppressive and pro-tumorigenic factors, underscoring its association with an active immunosuppressive landscape within the tumor microenvironment (Figure 5A,E). Gene set enrichment analysis (GSEA) reinforced these findings, demonstrating significant enrichment of pro-tumorigenic biological processes in the BTLA-high phenotype across TCGA and CGGA cohorts. These included the positive regulation of immunosuppressive cytokines (e.g., IL6 and IL10), pathways driving macrophage differentiation, and positive regulation of chemokine production (Figure 5B,F). Given that the BTLA-high microenvironment was transcriptionally characterized by cytokine signaling and myeloid cell enrichment, we hypothesized an association between BTLA and the phenotypic distribution of tumor-associated macrophages (TAMs). TAMs are a major component of the glioma TME, and their alternative M2 polarization is a hallmark of immunosuppression. Accordingly, we found that BTLA expression was significantly correlated with key molecular drivers of the M2 phenotype, including soluble factors and signaling molecules such as TGFβ, CSF1, IL6, IL10, STAT3, and IL13 (Figure 5C,G). To validate the impact of this signaling axis on the cellular landscape, we used the TIDE algorithm to estimate M2 macrophage infiltration. Consistent with our transcriptional findings, BTLA expression demonstrated a significant positive correlation with the TAM.M2 TIDE score in both TCGA (r = 0.14, *p* = 0.048) and CGGA (r = 0.15, *p* = 0.0069) cohorts (Figure 5D,H). Collectively, these results indicate that high BTLA expression was associated with an active transcriptional program that fosters an immunosuppressive niche.

### 3.5. BTLA Expression Was Enriched in Exhausted CD8^+^ T Cells and cDCs in the GBM Microenvironment

To translate these bulk transcriptomic associations of BTLA to the cellular level, we analyzed its expression across two independent GBM datasets. The first was a single-cell RNA-seq dataset (GSE131928_Smartseq2) from the TISCH2 database, which included 28 grade 4 GBM patients. The second consisted of two citeSeq datasets from the Brain Immune Atlas (Human GBM DCs citeSeq and Human GBM TAM, DCs and Monocytes citeSeq, *n* = 7), which incorporate protein-level detection.

**Figure 6:**
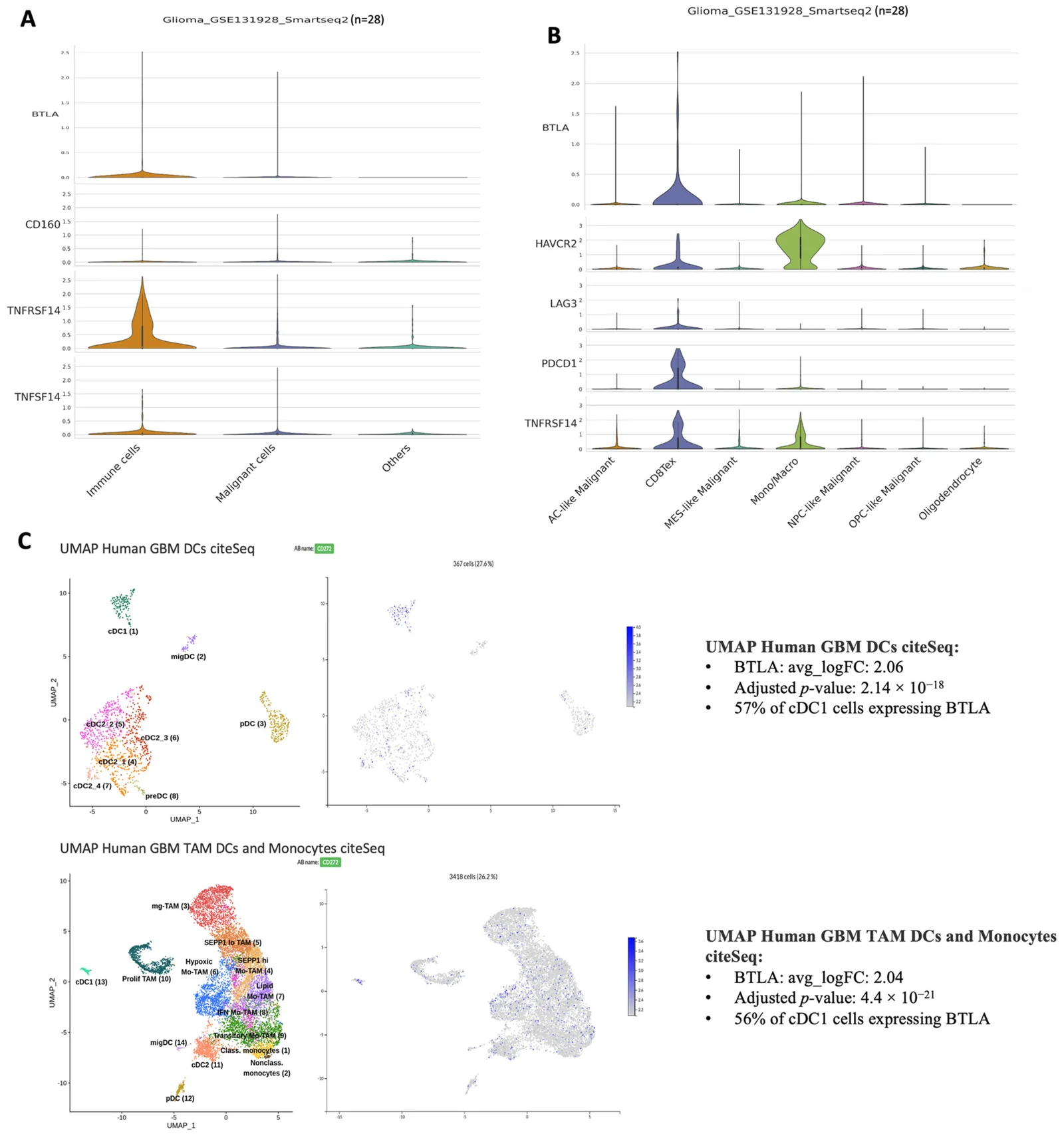
Single-cell multi-omic profiling of BTLA in the GBM microenvironment. (**A**) Single-cell expression and distribution. Violin plots (TISCH2, GSE131928_Smartseq2, *n* = 28) showing BTLA expression restricted to the immune compartments. Primary ligand HVEM (TNFRSF14) of BTLA was ubiquitously expressed across immune and malignant cells, while CD160 and LIGHT (TNFSF14), two alternative binding partners of HVEM, show no significant immune enrichment. (**B**) BTLA expression in exhausted CD8 T cells. Violin plots revealed BTLA expression in 12% of exhausted CD8 T cells (CD8Tex) (log2FC: 0.25, p-adj: 2.36 × 10^−40^), whereas HVEM (TNFRSF14) was present in 41.8% of CD8Tex cells (log2FC: 0.62, p-adj: 3.52 × 10^−12^), and PD-1 (PDCD1), detected in 41.8% of the CD8Tex population (log2FC: 1.01, p-adj: 2.68 × 10^−132^). Single-cell correlation analysis revealed positive correlations of BTLA with PD1 (r = 0.30), LAG3 (r = 0.28), and HAVCR2 (r = 0.22). (**C**) BTLA expression in myeloid subsets. UMAP projections from the Brain Immune Atlas (citeSeq) showing high-confidence protein-level detection of BTLA (CD272) specifically within the cDC1 cluster. Detection rates were 57% and 56% across Human GBM DCs citeSeq (*n* = 367 cells, top) and Human GBM TAMs, DCs, and monocytes citeSeq datasets (*n* = 3418 cells, bottom) (p-adj: 2.14 × 10^−18^ and 4.4 × 10^−21^, respectively).

Initial profiling of GSE131928_Smartseq2 revealed that BTLA expression was predominantly confined to immune cells (Figure 6A). The primary ligand, HVEM (TNFRSF14), exhibited ubiquitous expression across both malignant and immune cell populations (32.1% in MES-like malignant cells, p-adj: 2.5 × 10^−67^; 57% in macrophages, p-adj: 9.1 × 10^−36^; and 41.8% in CD8Tex, p-adj: 3.5 × 10^−12^) (Figure 6A,B). In contrast, the alternative HVEM binding partners CD160 and LIGHT (TNFSF14) were not significantly enriched in immune cells. This widespread availability of HVEM suggests a broad inhibitory potential for BTLA-positive infiltrates throughout the tumor microenvironment.

Further investigation into T cell states showed that BTLA was significantly enriched in exhausted CD8^+^ T cells (CD8Tex) (log2FC: 0.25, p-adj: 2.36 × 10^−40^), with a detection rate of 12%. Notably, this expression pattern mirrored that of the classical exhaustion marker PD-1 (PDCD1), which was detected in 41.8% of the CD8Tex population. In addition, single-cell correlation analysis revealed a positive correlation between BTLA and PD-1 (r = 0.30), LAG3 (r = 0.28), and HAVCR2 (TIM3, r = 0.22) (Figure 6B). These data indicated that the BTLA-HVEM axis is a core component of the multi-checkpoint exhaustion program in GBM-infiltrating lymphocytes.

Finally, we investigated BTLA expression in the myeloid compartment using citeSeq data from the Brain Immune Atlas. This multi-omic approach revealed the robust presence of BTLA (CD272) within the myeloid compartment. Specifically, we observed high-confidence detection of BTLA in the cDC1 (type 1 conventional dendritic cell) cluster, with detection rates of 57% and 56% across the two datasets (p-adj: 2.14 × 10^−18^ and 4.4 × 10^−21^, respectively) (Figure 6C). These findings suggest a potential link between BTLA and the regulation of both the effector T cell response and the antigen presentation phase via cDC1s in GBM.

### 3.6. BTLA Defined Dysfunctional CD8^+^ and CD4^+^ T Cells in the Peripheral Blood of GBM Patients, Characterized by Impaired IFN-γ Production and Reduced Cytotoxic Capacity

**Figure 7:**
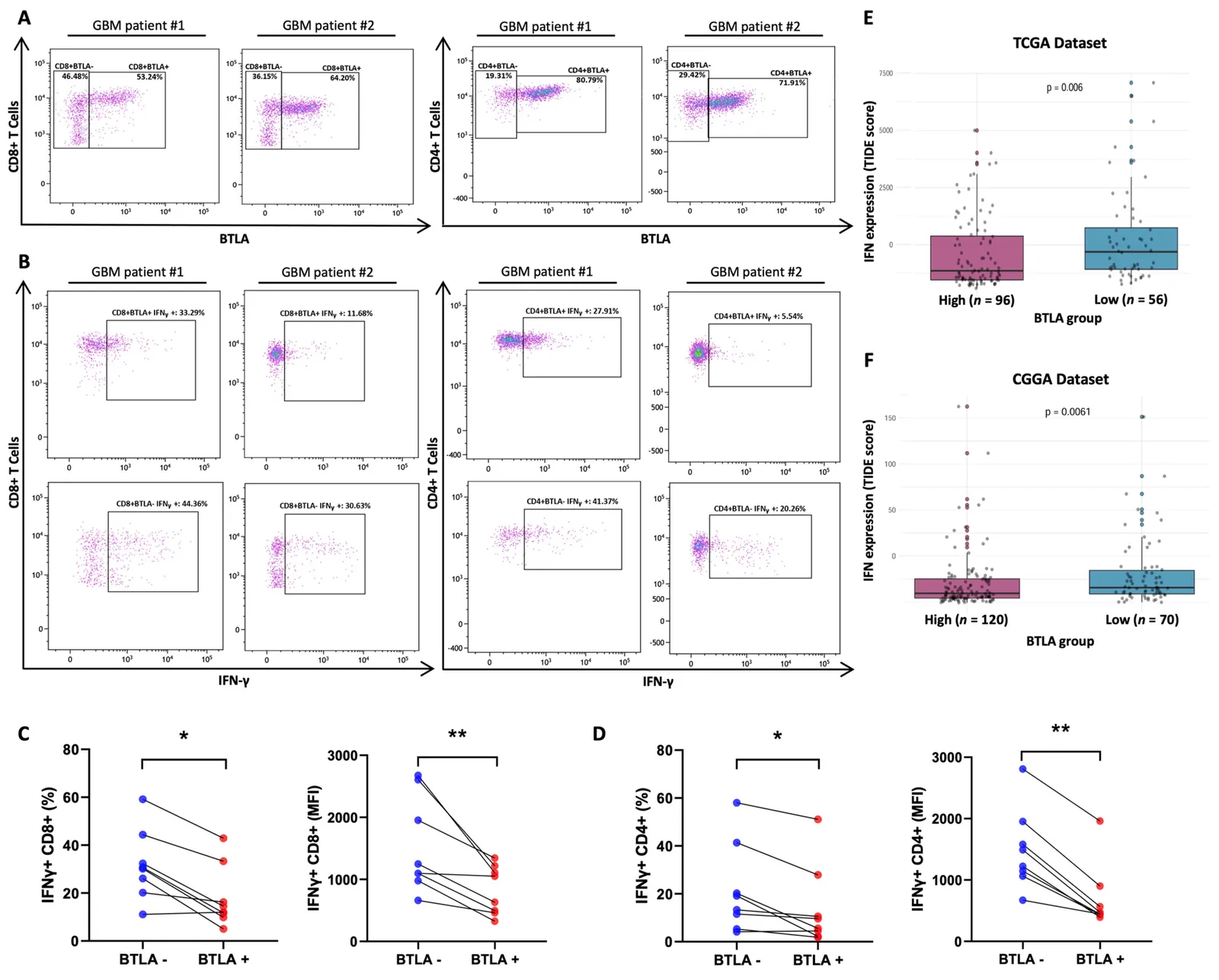
BTLA expression defined dysfunctional CD8^+^ and CD4^+^ T cells characterized by reduced IFN-γ production. (**A**) Phenotypic characterization: Representative flow cytometry plots illustrating BTLA protein expression on peripheral blood CD8^+^ (left) and CD4^+^ (right) T cells from two GBM patients (GBM patient #1 and #2). (**B**) Functional analysis: Representative flow cytometry plots of intracellular IFN-γ production induced by anti-CD3/CD28 in PBMCs from GBM patients. Plots are gated on BTLA^+^ and BTLA^−^ subsets within the CD8^+^ and CD4^+^ T cell compartments. (**C**,**D**) BTLA^−^ T cells exhibited significantly higher levels of IFN-γ production compared to BTLA^+^ counterparts. Line graphs (*n* = 8) comparing IFN-γ secretion between BTLA^−^ and BTLA^+^ subsets in CD8^+^ (**C**) and CD4^+^ (**D**) T cell populations. Significant reductions in IFN-γ were observed in the BTLA^+^ compartment across both frequency and mean fluorescence intensity (MFI) for CD8^+^ (freq: *p* = 0.0156, r = 0.54; MFI: *p* = 0.0078, r = 0.8) and CD4^+^ T cells (freq: *p* = 0.0156, r = 0.6; MFI: *p* = 0.0078, r = 0.78). (**E**,**F**) In silico validation: Boxplots showing the association between BTLA mRNA expression and IFN signature scores in large-scale GBM cohorts from TCGA (*n* = 152, (**E**)) and CGGA (*n* = 190, (**F**)) datasets. BTLA-high patients exhibited significantly lower IFN signatures compared to BTLA-low patients (TCGA: *p* = 0.006, r = 0.22; CGGA: *p* = 0.0061, r = 0.20). BTLA-low and high groups were classified using an optimal bootstrap cutoff. *p*-values were determined by a paired Wilcoxon Signed Rank test for (**C**,**D**) and a Mann–Whitney test for (**E**,**F**). Two-tailed *p*-value < 0.05 was considered significant; * *p*-value < 0.05; ** *p*-value < 0.01.

Building upon our single-cell observations that BTLA mRNA was enriched in exhausted T cell clusters and correlated with established checkpoint markers, we sought to validate these findings at the protein level and assess their functional consequences. Using flow cytometry to profile the peripheral blood mononuclear cells (PBMCs) of GBM patients, we confirmed that the BTLA protein was robustly expressed in both circulating CD8^+^ and CD4^+^ T cell subsets (Figure 7A).

To determine if this BTLA expression translates to effector dysfunction, we performed a functional assay by stimulating PBMCs with anti-CD3/CD28, followed by intracellular staining for IFN-γ. Representative plots gated on the BTLA^−^ and BTLA^+^ subsets within the CD8^+^ and CD4^+^ compartments (Figure 7B) revealed that BTLA^+^ T cells produce markedly less IFN-γ than their BTLA^−^ counterparts.

Quantitative assessment of the patient cohort (*n* = 8, Figure 7C,D) confirmed that this functional deficit was consistent across individuals. BTLA^−^ T cells exhibited significantly higher levels of IFN-γ production in terms of both the frequency of cytokine-secreting cells and the Mean Fluorescence Intensity (MFI). This significant reduction was evident in both CD8^+^ (freq: *p* = 0.0156, r = 0.54; MFI: *p* = 0.0078, r = 0.8) and CD4^+^ T cells (freq: *p* = 0.0156, r = 0.6; MFI: *p* = 0.0078, r = 0.78).

Finally, to bridge our clinical flow cytometry data back to large-scale transcriptomic cohorts, we performed TIDE analysis to evaluate the interferon (IFN) signature expression within the BTLA-low and BTLA-high groups. The TIDE IFN signature serves as a surrogate for the effector state of the tumor microenvironment. In alignment with our functional flow cytometry results, TIDE analysis revealed that patients classified as BTLA-high exhibited significantly lower IFN signature scores than those classified as BTLA-low in both TCGA (*n* = 152, *p* = 0.006, r = 0.22) and CGGA (*n* = 190, *p* = 0.0061, r = 0.20) GBM datasets (Figure 7E,F).

Collectively, these results demonstrate that BTLA expression defines both CD8^+^ and CD4^+^ T cells with impaired IFN-γ production, extending the BTLA-associated dysfunctional phenotype beyond cytotoxicity to include defective cytokine secretion.

**Figure 8:**
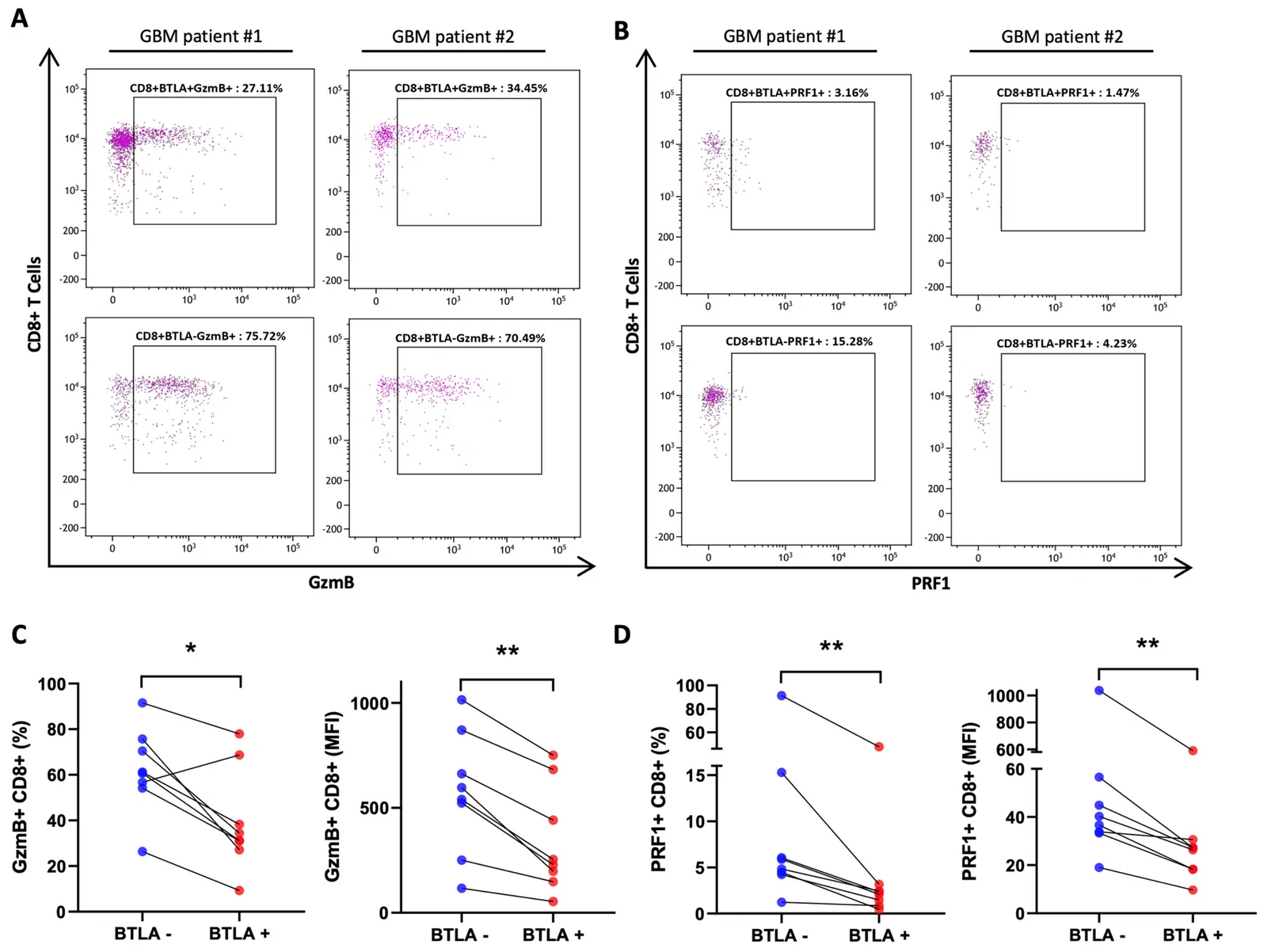
BTLA^+^CD8^+^ T cells exhibited impaired cytotoxic function characterized by reduced granzyme B and perforin expression. (**A**,**B**) Representative phenotyping: Flow cytometry plots of peripheral blood CD8^+^ T cells from two representative GBM patients (GBM patient #1 and #2) illustrating the production of granzyme B (GzmB, (**A**)) and perforin (PRF1, (**B**)). Plots are gated on BTLA^+^ and BTLA^−^ subsets within the CD8^+^ T cells. (**C**,**D**) Quantitative Assessment: Line graphs (*n* = 8) comparing the frequency (left) and MFI (right) of granzyme B (**C**) and perforin (**D**) between BTLA^−^ and BTLA^+^CD8^+^ T cell subsets. BTLA^+^CD8^+^ T cells exhibited significantly lower levels of both cytotoxic granules across both frequency of positive cells (GzmB: *p* = 0.0156, r = 0.5; PRF1: *p* = 0.0078, r = 0.8) and MFI (GzmB: *p* = 0.0078, r = 0.9; PRF1: *p* = 0.0078, r = 0.7). Statistical significance was determined by a paired Wilcoxon Signed Rank test. Two-tailed *p*-value < 0.05 was considered significant; * *p*-value < 0.05; ** *p*-value < 0.01.

Having established that BTLA^+^ T cells exhibit defective cytokine production, we next investigated whether BTLA expression also marks CD8^+^ T cells with impaired cytotoxic function. We analyzed the production of granzyme B (GzmB) and perforin (PRF1) using flow cytometry within the BTLA^+^CD8^+^ and BTLA^−^CD8^+^ T cell subsets. Representative data from two patients showed that BTLA^+^CD8^+^ T cells express lower levels of granzyme B and perforin than BTLA^−^CD8^+^ T cells (Figure 8A,B). Quantitative analysis across patients (*n* = 8, Figure 8C,D) confirmed that the BTLA^+^CD8^+^ T cell subsets exhibited significantly lower levels of granzyme B and perforin. Specifically, both the frequency and MFI of granzyme B and perforin were significantly reduced in BTLA^+^CD8^+^ T cells compared to those in BTLA^−^CD8^+^ T cells (GzmB: *p* = 0.0156, r = 0.5; PRF1: *p* = 0.0078, r = 0.7) and MFI (GzmB: *p* = 0.0078, r = 0.9; PRF1: *p* = 0.0078, r = 0.7).

Collectively, these findings demonstrate that BTLA expression on T cells was associated with multifaceted functional impairment, including reduced IFN-γ production in both CD8^+^ and CD4^+^ subsets and diminished cytotoxic capacity specifically within the CD8^+^ compartment. While these findings establish BTLA as a phenotypic marker of T cell dysfunction in GBM patients, whether BTLA directly drives this functional deficit or serves as a secondary counter-regulatory marker induced by chronic activation remains to be functionally dissected using targeted blockade models.

## 4. Discussion

In this study, we comprehensively analyzed the expression patterns and clinical relevance of BTLA in human glioma across three independent cohorts: the Moroccan cohort, TCGA, and CGGA datasets. To ensure a meaningful and consistent comparison between these datasets, our analytical strategy was centered on molecularly homogeneous groups that remain foundational to both WHO 2016 and WHO 2021 classifications. By prioritizing shared molecular entities, specifically IDH mutation status and histological grade, we were able to mitigate the biological heterogeneity inherent in glioma studies and evaluated three primary groups: GBM, IDH-wildtype, grade 4; Astrocytoma, IDH-mutant; and Oligodendroglioma.

Our findings revealed that BTLA expression was significantly upregulated in patients with IDH-wildtype status, a molecular signature strongly associated with poor clinical prognosis, compared to IDH-mutant tumors. Furthermore, BTLA levels are markedly elevated in GBM, representing the most malignant pathological subtype. We note that while statistical differences in BTLA expression were identified across histological types, grades, and IDH mutation statuses across all three cohorts, a low *p*-value does not inherently denote clinical meaningfulness. In large public databases such as TCGA and CGGA, high statistical power can render subtle relative shifts in mRNA expression statistically significant. From a translational perspective, a clinically meaningful difference in BTLA expression translates to functional immunosuppression; namely, sufficient receptor density at the immunological synapse to recruit SHP-1/2, inhibit TCR signaling, and impact patient overall survival. While our local cohort demonstrates a pronounced separation in magnitude between low-grade/IDH-mutant tumors and IDH-wildtype GBM, functional cellular validation and clinical outcome correlations remain essential to confirm the biological impact of these statistical differences. This result is particularly significant in light of the work by Han et al., which demonstrated that HVEM, the primary ligand for BTLA, was also strongly upregulated in aggressive gliomas, specifically within the IDH-wildtype GBM subgroup, and correlated with worse patient outcomes [25]. The concurrent dysregulation of this ligand-receptor strongly suggests that the BTLA/HVEM inhibitory signaling pathway plays a key role in mediating immunosuppression within the glioma microenvironment. Collectively, our results indicate that elevated BTLA expression is associated with an aggressive phenotype and features of glioma malignancy. This role is not unique to glioma but is consistent across a spectrum of malignancies. In solid tumors such as gastric cancer [26], lung cancer [27], epithelial ovarian carcinoma [28], and hematologic malignancies such as chronic and acute myeloid leukemia [29,30], BTLA expression drives T-cell dysfunction and serves as a marker of aggressive disease and poor prognosis. Mirroring these reports, our work confirms that high BTLA expression in the glioma microenvironment was correlated with poor overall survival in univariate analysis across both datasets. However, its independent prognostic value was cohort-dependent, reaching significance in the larger CGGA cohort but not in TCGA. This statistical discrepancy is likely driven by the smaller sample size of the TCGA cohort and the strong confounding influence of IDH status and tumor grade, clinical features with which BTLA expression is biologically and tightly associated. Moreover, we found that co-expression of BTLA and PD-1 predicted an even more dismal prognosis across both datasets. This double-high phenotype led to a significant survival disadvantage in CGGA and a striking six-fold increased risk of death in TCGA compared to the double-low reference group. The positive correlation between BTLA and PD-1, combined with their cooperative association with poor survival, suggests that these inhibitory pathways may act together to promote immune evasion in glioma, highlighting BTLA as a potent marker of aggressive disease and a promising candidate for dual checkpoint targeted therapies. This cooperative inhibitory function was conserved across cancers, as demonstrated in NSCLC, where BTLA and PD-1 synergistically suppress CD8^+^ T cell activity and contribute to resistance against PD-1/PD-L1 immunotherapy [31]. Importantly, the co-inhibition of BTLA and PD-L1 enhanced antitumor efficacy, suggesting a promising combinatorial strategy [31]. This co-expression pattern extends to a wide range of other immune checkpoints (PD-L1, TIM3, CTLA-4, VISTA, LAG3, and B7-H3), indicating that BTLA may be part of a broader network of compensatory and redundant immunosuppressive pathways.

As monotherapy targeting a specific immune checkpoint has proven ineffective in treating GBM [32], immunotherapy combined with other targets may lead to the development of new therapeutic strategies to effectively inhibit GBM growth and enhance the antitumor response and survival of patients with advanced glioma [4,33].This approach is strongly supported by preclinical evidence. In a GBM murine model, dual PD-1 and BTLA blockade synergistically enhanced survival by 60% and boosted IFN-γ production in T cells [34]. Thus, our data corroborate these functional findings and provide a compelling rationale for prioritizing BTLA inhibition as a component of combination immunotherapy with PD-1/PD-L1 blockade in human gliomas.

An in-depth understanding of the tumor microenvironment will contribute to the development of more effective treatments and improve patient prognosis [35]. Our ESTIMATE analysis revealed abundant stromal and immune infiltration and lower tumor purity in the BTLA-high phenotype. In gliomas, low tumor purity has been established as an independent predictor of poor prognosis, cellular heterogeneity, and aggression [36]. Moreover, immune cell analysis revealed that high BTLA expression identified a TME enriched with both potent effector populations and multifaceted immunosuppressive subsets. Within the CD8^+^ T cell compartment, there was simultaneous enrichment of central memory and cytotoxic cells along with exhausted subsets. In addition, the BTLA-high TME was heavily infiltrated by multiple regulatory T cell lineages (Tr1, nTreg, and iTreg) and diverse innate compartments, including macrophages, monocytes, dendritic cells, and NK cells. The coexistence of armed effector cells with potent inhibitory activity creates a paradox. One interpretation is that the TME may not be immune-deserted but rather actively suppressed, a state in which the antitumor potential of recruited effectors may be systematically neutralized by a dominant regulatory network. This interpretation is inferential and supported by spatial distribution analyses. A recent multispectral fluorescent imaging study conducted by Wang et al. demonstrated that in GBM, Tregs are located closer to cytotoxic CD8^+^ T cells, particularly PD-1^+^CD8^+^ T cells, suggesting a targeted suppressive influence on the effector immune response [37]. Evidence from a GBM murine model also demonstrated a specific role for BTLA in Treg infiltration. Treatment with anti-BTLA monotherapy decreased Treg infiltration in the brain, whereas anti-PD-1 did not. This indicated that the reduction in Tregs observed with combination therapy was a direct effect of BTLA inhibition rather than PD-1 blockade [34]. This mechanism likely underpins the dysfunctional immune state and poor prognosis associated with high BTLA expression in the glioma microenvironment. Our results further substantiate this, showing that BTLA expression correlates significantly with Foxp3 Treg master regulator, potent inhibitory mediators (TGF-β and IL-10), and chemokines responsible for recruiting immunosuppressive cells. This was mechanistically supported by GSEA analysis, which showed enrichment of pathways regulating immunosuppressive cytokine and chemokine production. Beyond the lymphoid compartment, BTLA was positively correlated with master drivers of M2 macrophage polarization and a high infiltration of TAM.M2 (TIDE score) within the TME. Clinically, the immunosuppressive landscape of GBM, defined by Treg and M2 macrophage enrichment, is a major driver of treatment failure. Tregs confer resistance to VEGF therapy [38], while M2 macrophages promote radio-resistance [39], with both populations contributing to poor clinical outcomes.

Interestingly, to translate these bulk associations to the cellular level, we performed single-cell and citeSeq analyses, which revealed that BTLA expression was restricted to immune cells and was notably enriched in exhausted CD8^+^ T cells, where it correlated positively with PD-1, LAG3, and TIM3, positioning BTLA within the core multi-checkpoint exhaustion network in GBM. Its primary ligand, HVEM, was ubiquitously expressed across malignant and immune populations, suggesting a broad inhibitory potential. This aligns with studies on lung cancer and colorectal cancer, where BTLA co-expression defines an exhausted T cell phenotype associated with aggressive disease and poor prognosis [20,40,41]. Thus, our results provide evidence that the BTLA-HVEM axis may be involved in the dysfunction pathway of cytotoxic T cells in GBM. However, it remains to be determined whether this axis actively drives the impairment of effector functions or reflects a downstream feature of the overall exhausted immune state. Importantly, multi-omic citeSeq analysis extended these findings to the myeloid compartment, revealing high-confidence BTLA (CD272) expression on conventional type 1 dendritic cells (cDC1). Although BTLA is known to be expressed in both lymphoid and myeloid lineages, its presence on cDC1s has functional implications, as prior work has shown that BTLA^+^ cDC1s promote peripheral Treg induction in models of acute encephalomyelitis [42]. Similarly, Zhang et al. reported that BTLA^+^ cDC1s promote Treg and Th2 polarization of T cells in human lung tuberculosis [43].

Crucially, this immunosuppressive signature was not confined to the local tumor site, but extended to the systemic circulation. We demonstrated that BTLA protein expression identifies subsets of circulating CD8^+^ and CD4^+^ T cells that were characterized by significant functional impairment. BTLA^+^ T cells produced markedly less IFN-γ and BTLA^+^ CD8^+^ T cells exhibited diminished granzyme B and perforin expression. These protein-level findings were corroborated by TIDE analysis of large transcriptomic cohorts, which showed that BTLA-high GBM patients displayed significantly lower interferon signature scores in both the TCGA and CGGA datasets. It is important to contextualize that cross-sectional human tissue profiling establishes a phenotypic association rather than direct temporal causality, although functional recovery following BTLA blockade has been reported in a preclinical GBM model [34].

Our study is among the first to comprehensively demonstrate an association between BTLA expression and aggressive glioma biology across multiple independent human datasets. This study had several limitations. First, while our retrospective multi-cohort analysis (Moroccan, TCGA, CGGA) yielded strong correlative evidence, prospective studies in larger, expanded cohorts, particularly for the Moroccan population, are essential to confirm causality and generalizability. In addition, the substantial amount of missing clinical data, particularly regarding IDH mutation status and histological type, may have limited our ability to fully stratify the tumor microenvironment by these critical subtypes. Nevertheless, the distribution of available cases remained consistent with larger cohorts TCGA and CGGA, supporting the representativeness of our findings. These results should therefore be interpreted with caution, and future studies with complete clinical annotation are warranted to validate them in molecularly defined subgroups. Second, while single-cell and citeSeq analyses pinpointed BTLA expression in cDC1s and CD8Tex, a novel observation that warrants independent validation in multicenter cohorts, our data remain correlative. Consequently, we cannot definitively conclude whether BTLA actively contributes to T cell dysfunction or merely serves as a marker associated with an exhausted immune phenotype. Mechanistic characterization of BTLA’s role in Treg induction and T cell exhaustion was deliberately reserved for future investigations. Third, loss-of-function experiments in human glioma models, complementary to the present work, represent the logical next step to validate the cooperative association between BTLA/HVEM and PD-1/PD-L1 blockade, strongly supported by our data and prior murine evidence [34]. Importantly, while our flow cytometry analysis of circulating T cells was limited to a small cohort of eight GBM patients, these protein-level findings suggested systemic immune dysfunction as a potential biomarker; future paired analyses with tumor-infiltrating lymphocytes and localized proteomics in larger patient cohorts will clarify whether circulating BTLA^+^ T cells and public transcriptomic datasets truly reflect the intratumoral protein landscape.

## Data Availability

Data of Moroccan glioma patients presented in this study are not publicly available due to ethical and privacy restrictions, but are available upon request from the corresponding author.

## Supplementary Materials

The following supporting information can be downloaded at: https://www.mdpi.com/article/10.3390/medsci14040433/s1. Supplementary Table S1. BTLA gene expression across different clinicopathological features in Moroccan cohort. Supplementary Table S2. BTLA gene expression across different clinicopathological features in TCGA and CGGA datasets. Supplementary Figure S1. Multivariate Cox analysis of BTLA expression and clinicopathological factors in TCGA and CGGA. Forest plots showing the independent prognostic value of BTLA expression along with other clinical variables, including age, tumor garde, IDH mutation status, and gender (sex), in (A) 276 patients from TCGA and (B) 609 from CGGA cohorts. Hazard ratios (HRs) with 95% CIs and two-sided *p*-values are displayed for each covariate. After adjusting for potential confounders, elevated BTLA expression remained a significant independent predictor of poorer survival in the CGGA cohort (HR = 1.7, 95% CI: 1.24–2.3, *p* < 0.001), whereas no statistically significant independent association was observed in the TCGA cohort (HR = 1.2, 95% CI: 0.85–1.8, *p* = 0.267). Patients were classified into low and high BTLA expression groups based on an optimal bootstrap validated cutoff. * *p* < 0.05; ** *p* < 0.01; *** *p* < 0.001. Supplementary Figure S2. Multivariate Cox analysis of PD-1 expression and clinicopathological factors in TCGA and CGGA. Forest plots showing the independent prognostic value of PD-1 expression along with other clinical variables, including age, tumor garde, IDH mutation status, and gender (sex), in (A) 276 patients from TCGA and (B) 609 from CGGA cohorts. Hazard ratios (HRs) with 95% CIs and two-sided *p*-values are displayed for each covariate. After adjusting for potential confounders, elevated PD-1 expression remained a significant independent predictor of poorer survival in the CGGA cohort (HR = 2.0, 95% CI: 1.1–2.0, *p* = 0.01), whereas no statistically significant independent association was observed in the TCGA cohort (HR = 1.1, 95% CI: 0.8–2.0, *p* = 0.49). Patients were classified into low and high PD-1 expression groups based on an optimal bootstrap validated cutoff. * *p* < 0.05; ** *p* < 0.01; *** *p* < 0.001. Supplementary Figure S3. Multivariate Cox analysis of combined BTLA and PD-1 co-expression and clinicopathological factors in TCGA and CGGA. Forest plots showing the independent prognostic value of the double-high BTLA/PD-1 group along with other clinical variables, including age, tumor garde, IDH mutation status, and gender (sex), in (A) 276 patients from TCGA and (B) 609 from CGGA cohorts. Hazard ratios (HRs) with 95% CIs and two-sided *p*-values are displayed for each covariate. After adjusting for potential confounders, double-high BTLA/PD-1 expression remained a significant independent predictor of poorer survival in the CGGA cohort (HR = 3.0, 95% CI: 1.5–7.0, *p* = 0.0), whereas no statistically significant independent association was observed in the TCGA cohort (HR = 1.0, 95% CI: 0.6–2.0, *p* = 0.89). Patients were classified into combined low-BTLA/low-PD-1 and high-BTLA/high-PD-1 expression groups based on an optimal bootstrap validated cutoff. * *p* < 0.05; ** *p* < 0.01; *** *p* < 0.001. Supplementary Figure S4. Association between BTLA expression and tumor infiltration in the CGGA dataset. (A–D) Immune cell abundance: Violin plots illustrating immune cell distributions analyzed using ImmuCellAI algorithm, comparing BTLA-high (*n* = 145, red) and BTLA-low (*n* = 164, blue) phenotypes across (A) CD8+ T cells, (B) CD4+ T cells, (C) Treg subpopulations, and (D) general immune populations. (E) Heatmap of tumor microenvironment scores according to BTLA expression status using the ESTIMATE algorithm. Data are presented as mean values per group: BTLA-low (*n* = 164), BTLA-high (*n* = 145). Values represent raw means, and color denotes Z-score transformation (red: above mean; Blue: below mean). All scores were significantly elevated in the BTLA-high group (*p* < 0.0001) (F) Violin plots showing that BTLA-high tumors exhibited significantly lower tumor purity compared to the BTLA-low group (*p* < 0.0001). (G) Spearman correlation plots demonstrating positive correlation between BTLA expression and TGFβ (r = 0.37, *p* = 1.5 × 10^−11^), IL10 (r = 0.34, *p* = 5.4 × 10^−10^), and Foxp3 (r = 0.22, *p* = 7.1 × 10^−5^). Group comparisons were performed using the Mann-Whitney test (A–D,F) or Welch’s *t*-test (E). A two-tailed *p*-value < 0.05 was considered significant, ns, not significant; * *p* < 0.05; ** *p* < 0.01; *** *p* < 0.001; **** *p* < 0.0001.

## Abbreviations

BTLA, B and T Lymphocyte Attenuator; CNS, Central Nervous System; CGGA, Chinese Glioma Genome Atlas; CTLA-4, Cytotoxic T-lymphocyte-associated protein 4; FDA, Food and Drugs Administration; GBM, Glioblastoma; GSEA, Gene Set Enrichment Analysis; HVEM, Herpes Virus Entry Mediator; IDH, Isocitrate Dehydrogenase; IFN-γ, Interferon gamma; IL-10, Interleukin 10; IL-6, Interleukin-6; LAG-3, Lymphocyte-activation gene 3; LGG, Low-Grade Glioma; MAIT, Mucosal-Associated Invariant T cell; MDSC, Myeloid-derived suppressor cells; NES, Normalized Enrichment Score; NK, Natural killer cell; NKT, Natural killer T cell; PD-1, Programmed cell death 1; PD-L1, Programmed death-ligand 1; TCGA, The Cancer Genome Atlas; TGF-β, Transforming growth factor-β; TIGIT, T-cell immunoglobulin and ITIM domain; TIICs, Tumor-Infiltrating Immune Cells; TILs, Tumor-Infiltrating Lymphocytes; TIM-3, T cell Immunoglobulin and Mucin domain- containing protein 3; TISCH2, Tumor Immune Single-cell Hub2; TME, Tumor Microenvironment; UMAP, Uniform Manifold Approximation and Projection; VEGF, Vascular endothelial growth factor; VISTA, V-domain Ig suppressor of T cell activation; WHO, World Health Organization; WHO CNS5, 5th edition of the WHO classification of Tumors of the Central Nervous System.
