# Supplementary material for: Elevated BTLA Expression Correlates with an Immunosuppressive Microenvironment and Defines Dysfunctional Circulating T Cells in Human Glioblastoma
Source: Med Sci (Basel). 2026 Jul 25;14(4):433. doi: 10.3390/medsci14040433 (PMC13515439; doi:10.3390/medsci14040433)
Supplement: Supplementary file 1 [file medsci-14-00433-s001.zip › medsci-4415769-supplementary.pdf]

Supplementary Table S1. BTLA gene expression across different clinicopathological features in Moroccan cohort

Clinicopathological parameters	Cases (%) (*n* = 44)	*p*-value
**Age**		
▪ **<= 45**	25 (56.8 %)	
▪ **> 45**	19 (43.2 %)	0.0252
**Sex**		
▪ **Male**	27 (61.4 %)	
▪ **Female**	17 (38.6 %)	0.6291
**WHO grade**		
▪ **Grade 1**	14 (31.8 %)	
▪ **Grade 2**	5 (11.4 %)	
▪ **Grade 3**	3 (6.8 %)	
▪ **Grade 4**	22 (50 %)	0.0020
**IDH mutation status**		
▪ **Yes**	8 (18.2 %)	
▪ **No**	16 (36.4 %)	0.0087
▪ Missing data	20 (45.4 %)	
**Histological type**		
▪ **Glioblastoma IDH-wildtype**	16 (32.7 %)	0.0101
▪ **Astrocytoma IDH-mutant**	8 (16.3 %)	
▪ **Oligodendroglioma**	3 (6.1 %)	
▪ Missing data	17 (44.9 %)	

***Statistical analyses were performed on bold parameters using the Mann-Whitney test for two independent groups and the Kruskal-Wallis test for multiple groups, followed by Dunn's multiple comparison test.**

Supplementary Table S2. BTLA gene expression across different clinicopathological features in TCGA and CGGA datasets

TCGA Dataset			CGGA Dataset		
**Clinicopathological parameters**	**Cases (%) (*n* = 667)**	***p*-value**	**Clinicopathological parameters**	**Cases (%) (*n* = 693)**	***p*-value**
**Age median**			**Age median**		
▪ **<= 46**	340 (51 %)		▪ **<= 43**	363 (52.4 %)	
▪ **> 46**	327 (49 %)	0.5817	▪ **> 43**	329 (47.5 %)	0.6148
			▪ Missing data	1 (0.1 %)	
**Sex**			**Sex**		
▪ **Male**	384 (57.6 %)		▪ **Male**	398 (57.4 %)	
▪ **Female**	283 (42.4 %)	0.0240	▪ **Female**	295 (42.6 %)	0.0840
**WHO grade**			**WHO grade**		
▪ **Grade 2**	249 (37.3 %)		▪ **Grade 2**	188 (27.1 %)	
▪ **Grade 3**	265 (39.7 %)		▪ **Grade 3**	255 (36.8 %)	
▪ **Grade 4**	152 (22.8 %)	0.0004	▪ **Grade 4**	190 (27.4 %)	0.0008
▪ Missing data	1 (0.2 %)		▪ Missing data	60 (8.7 %)	
**IDH mutation status**			**IDH mutation status**		
▪ **Yes**	63 (9.4 %)		▪ **Yes**	142 (20.5 %)	
▪ **No**	152 (22.8 %)	0.0009	▪ **No**	190 (27.4 %)	0.0239
▪ Missing data	452 (67.8 %)		▪ Missing data	361 (52.1 %)	
**Histological type**			**Histological type**		
▪ **Glioblastoma IDH-wildtype**	152 (22.8 %)	<0.0001	▪ **Glioblastoma IDH-wildtype**	190 (27.4 %)	0.0008
▪ **Astrocytoma IDH-mutant**	32 (4.8 %)		▪ **Astrocytoma IDH-mutant**	84 (12.1 %)	
▪ **OD**	31 (4.6 %)		▪ **OD**	58 (8.4 %)	
▪ Other and Missing data	452 (67.8 %)		▪ Other and Missing data	361 (52.1 %)	

OD: Oligodendroglioma. *Statistical analyses were performed on bold parameters using the Mann-Whitney test for two independent groups and the Kruskal-Wallis test for multiple groups, followed by Dunn's multiple comparison test.

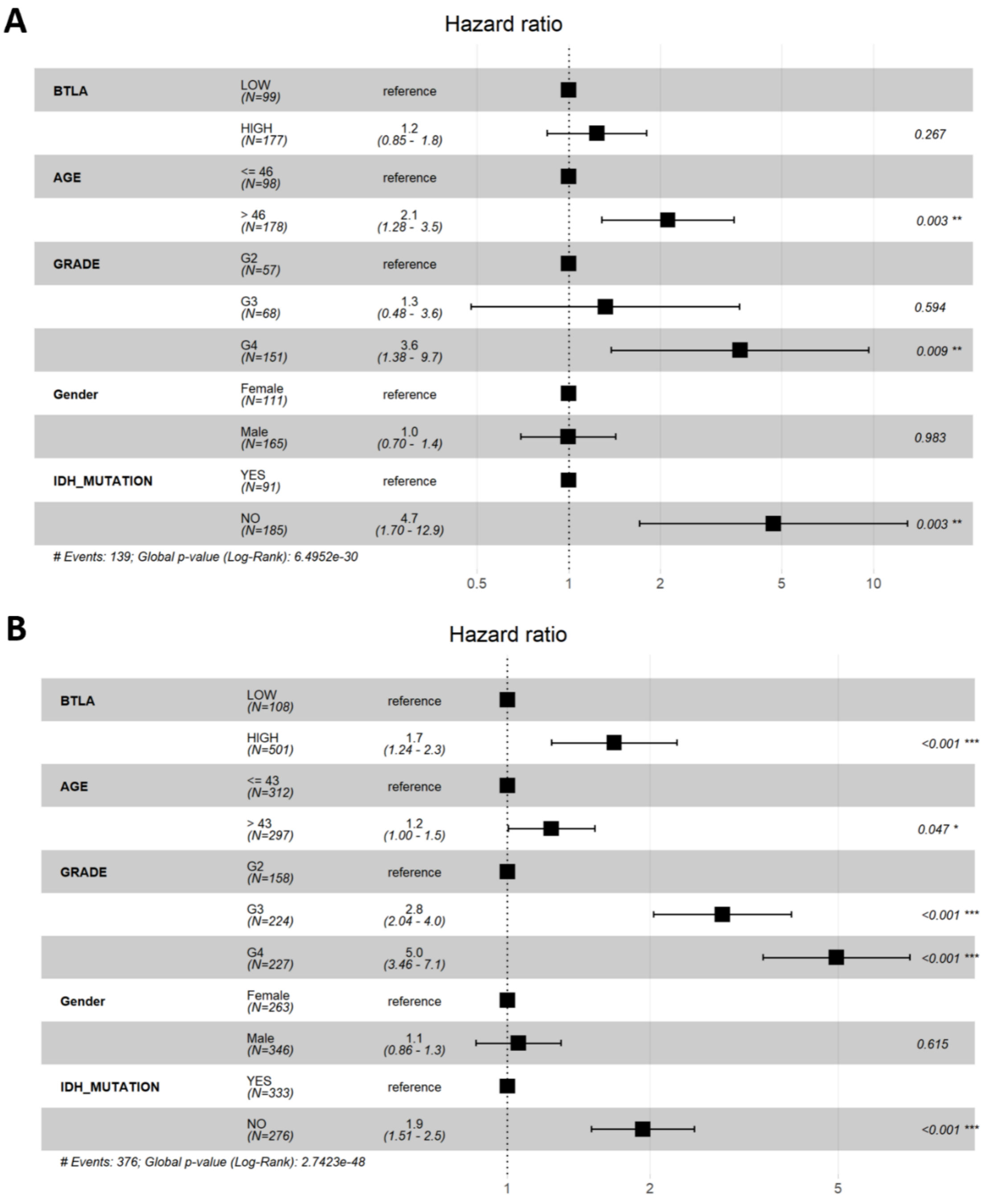

Supplementary Figure S1. Multivariate Cox analysis of BTLA expression and clinicopathological factors in TCGA and CGGA. Forest plots showing the independent prognostic value of BTLA expression along with other clinical variables, including age, tumor garde, IDH mutation status, and gender (sex), in **(A)** 276 patients from TCGA and **(B)** 609 from CGGA cohorts. Hazard ratios (HRs) with 95% CIs and two-sided *p*-values are displayed for each covariate. After adjusting for potential confounders, elevated BTLA expression remained a significant independent predictor of poorer survival in the CGGA cohort (HR = 1.7, 95% CI: 1.24-2.3, $p < 0.001$), whereas no statistically significant independent association was observed in the TCGA cohort (HR = 1.2, 95% CI: 0.85-1.8, $p = 0.267$). Patients were classified into low and high BTLA expression groups based on an optimal bootstrap validated cutoff. *$p < 0.05$; **$p < 0.01$; ***$p < 0.001$.

A

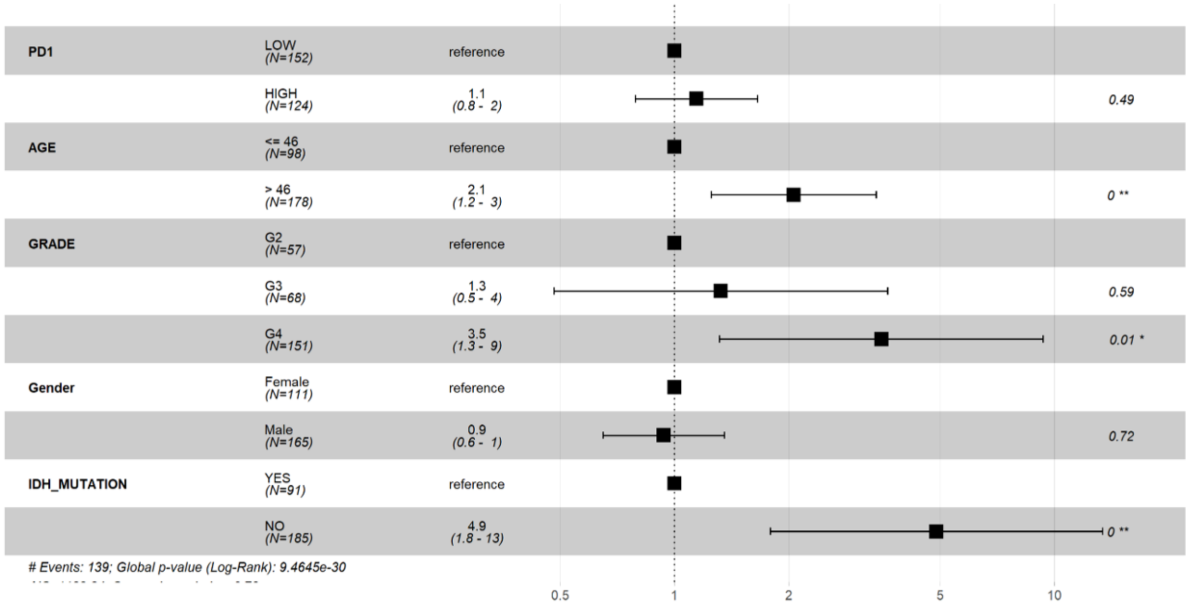

B

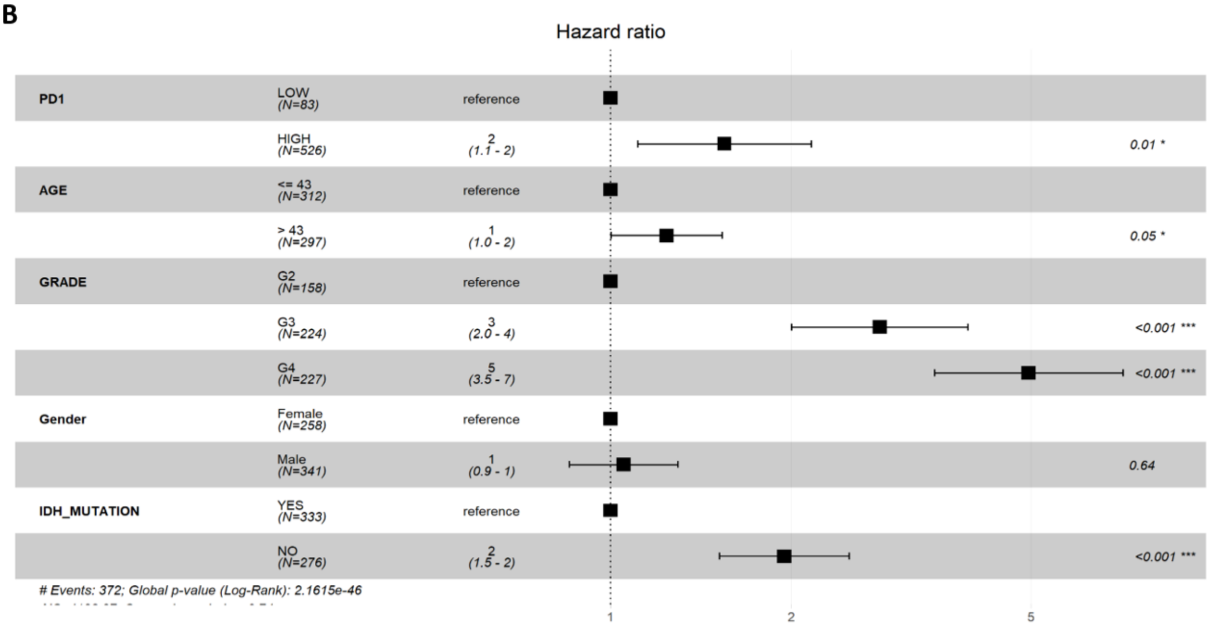

Supplementary Figure S2. Multivariate Cox analysis of PD-1 expression and clinicopathological factors in TCGA and CGGA. Forest plots showing the independent prognostic value of PD-1 expression along with other clinical variables, including age, tumor garde, IDH mutation status, and gender (sex), in **(A)** 276 patients from TCGA and **(B)** 609 from CGGA cohorts. Hazard ratios (HRs) with 95% CIs and two-sided *p*-values are displayed for each covariate. After adjusting for potential confounders, elevated PD-1 expression remained a significant independent predictor of poorer survival in the CGGA cohort (HR = 2.0, 95% CI: 1.1-2.0, $p = 0.01$), whereas no statistically significant independent association was observed in the TCGA cohort (HR = 1.1, 95% CI: 0.8-2.0, $p = 0.49$). Patients were classified into low and high PD-1 expression groups based on an optimal bootstrap validated cutoff. *$p < 0.05$; **$p < 0.01$; ***$p < 0.001$.

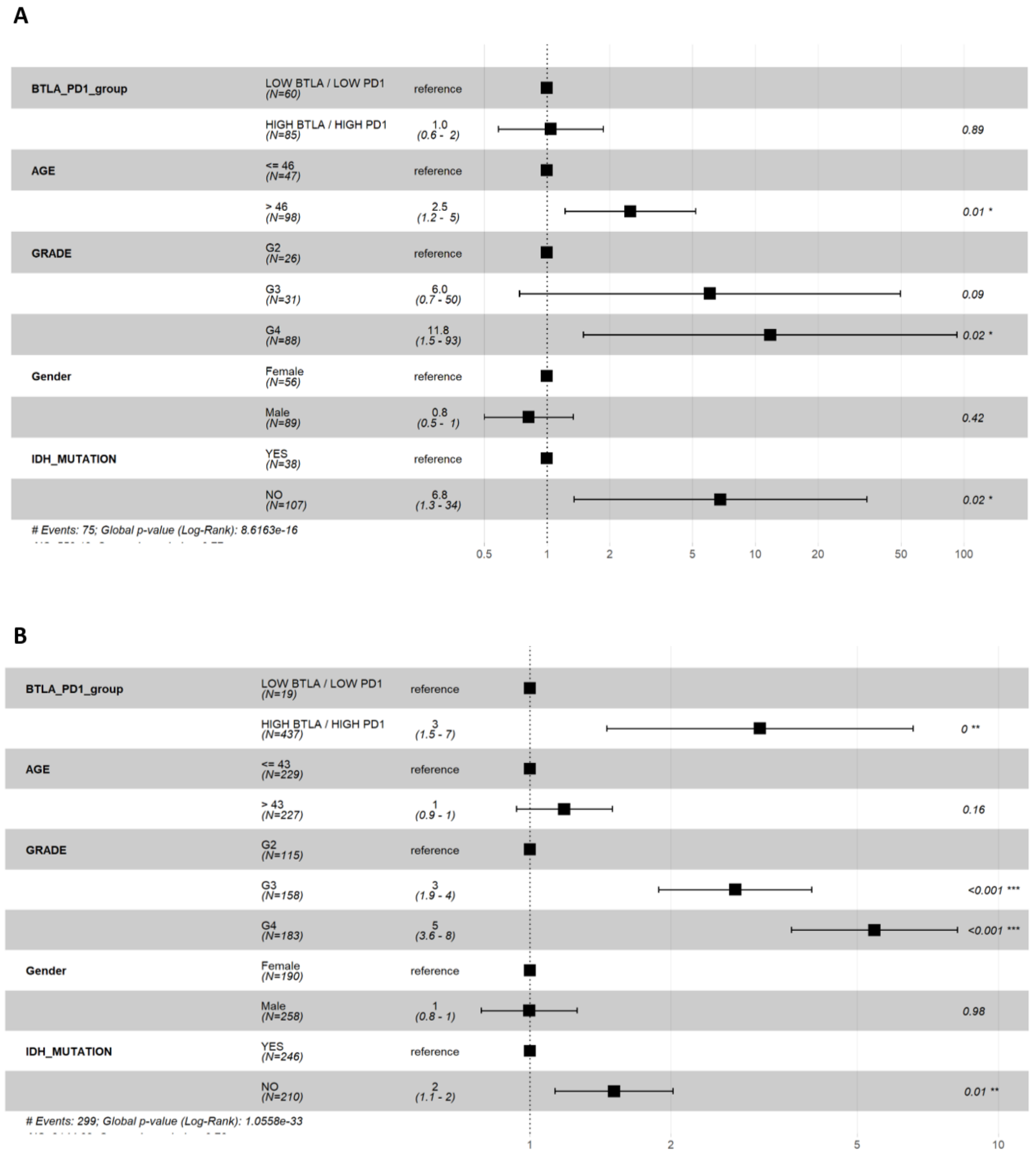

Supplementary Figure S3. Multivariate Cox analysis of combined BTLA and PD-1 co-expression and clinicopathological factors in TCGA and CGGA. Forest plots showing the independent prognostic value of the double-high BTLA/PD-1 group along with other clinical variables, including age, tumor garde, IDH mutation status, and gender (sex), in **(A)** 276 patients from TCGA and **(B)** 609 from CGGA cohorts. Hazard ratios (HRs) with 95% CIs and two-sided *p*-values are displayed for each covariate. After adjusting for potential confounders, double-high BTLA/PD-1 expression remained a significant independent predictor of poorer survival in the CGGA cohort (HR = 3.0, 95% CI: 1.5-7.0, p = 0.0), whereas no statistically significant independent association was observed in the TCGA cohort (HR = 1.0, 95% CI: 0.6-2.0, p = 0.89). Patients were classified into combined low-BTLA/low-PD-1 and high-BTLA/high-PD-1 expression groups based on an optimal bootstrap validated cutoff. *$p < 0.05$; **$p < 0.01$; ***$p < 0.001$.

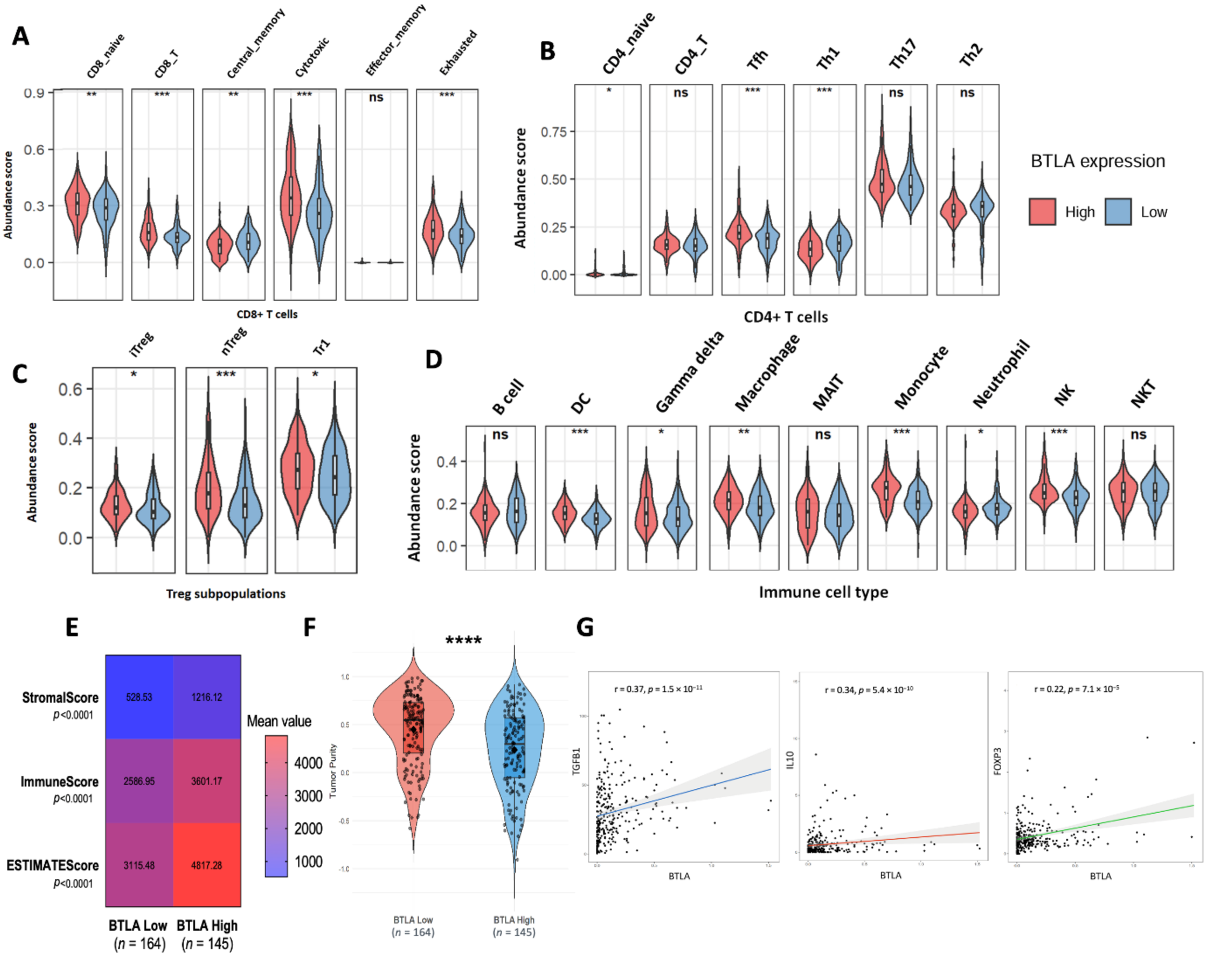

Supplementary Figure S4. Association between BTLA expression and tumor infiltration in the CGGA dataset. **(A-D)** Immune cell abundance: Violin plots illustrating immune cell distributions analyzed using ImmuCellAI algorithm, comparing BTLA-high (n = 145, red) and BTLA-low (n = 164, blue) phenotypes across **(A)** $CD8^+$ T cells, **(B)** $CD4^+$ T cells, **(C)** Treg subpopulations, and **(D)** general immune populations. **(E)** Heatmap of tumor microenvironment scores according to BTLA expression status using the ESTIMATE algorithm. Data are presented as mean values per group: BTLA-low (n = 164), BTLA-high (n = 145). Values represent raw means, and color denotes Z-score transformation (red: above mean; Blue: below mean). All scores were significantly elevated in the BTLA-high group ($p < 0.0001$) **(F)** Violin plots showing that BTLA-high tumors exhibited significantly lower tumor purity compared to the BTLA-low group ($p < 0.0001$). **(G)** Spearman correlation plots demonstrating positive correlation between BTLA expression and TGFβ ($r = 0.37$, $p = 1.5 \times 10^{-11}$), IL10 ($r = 0.34$, $p = 5.4\times 10^{-10}$), and Foxp3 ($r = 0.22$, $p = 7.1\times 10^{-5}$). Group comparisons were performed using the Mann-Whitney test **(A-D,F)** or Welch's t-test **(E)**. A two-tailed p-value < 0.05 was considered significant, ns, not significant; *$p < 0.05$; **$p < 0.01$; ***$p < 0.001$; ****$p < 0.0001$.
